# Instruction in information structuring improves Bayesian judgment in intelligence analysts

**DOI:** 10.3389/fpsyg.2015.00387

**Published:** 2015-04-08

**Authors:** David R. Mandel

**Affiliations:** Socio-Cognitive Systems Section, Defence Research and Development Canada and Department of Psychology, York UniversityToronto, ON, Canada

**Keywords:** instructional methods, Bayesian judgment, probability judgment, information structuring, coherence

## Abstract

An experiment was conducted to test the effectiveness of brief instruction in information structuring (i.e., representing and integrating information) for improving the coherence of probability judgments and binary choices among intelligence analysts. Forty-three analysts were presented with comparable sets of Bayesian judgment problems before and immediately after instruction. After instruction, analysts' probability judgments were more coherent (i.e., more additive and compliant with Bayes theorem). Instruction also improved the coherence of binary choices regarding category membership: after instruction, subjects were more likely to invariably choose the category to which they assigned the higher probability of a target's membership. The research provides a rare example of evidence-based validation of effectiveness in instruction to improve the statistical assessment skills of intelligence analysts. Such instruction could also be used to improve the assessment quality of other types of experts who are required to integrate statistical information or make probabilistic assessments.

## Introduction

Categorization under uncertainty is a basic fact of life. In a wide range of contexts, both personal and professional, people strive to accurately categorize “objects,” including, at times, themselves. Yet in many, if not most, cases, the correct category to which an object belongs is not immediately apparent. Instead, one might have to generate hypotheses about putative category membership. Moreover, the evidence one has at one's disposal is usually inconclusive, serving at best to amplify or attenuate support for the hypotheses under consideration. In other words, the evidence may not fully eliminate uncertainty about category membership yielding a definitive answer. Indeed, it is primarily because most everyday judgment and reasoning is made under conditions of uncertainty that the dominant normative paradigm for assessing reasoning quality has shifted from a truth functional logic of certain deduction to a Bayesian logic of uncertain deduction (e.g., Oaksford and Chater, 2007; Evans, 2012; Baratgin et al., 2014).

The literature on Bayesian reasoning is rich and the focus of this paper is restricted to two aspects of it: Bayes theorem and the complementarity constraint (Baratgin and Noveck, 2000), which is a special case of the axiom of finite additivity of closed subsets, often called the additivity principle in cognitive psychology (e.g., Tversky and Koehler, 1994; Villejoubert and Mandel, 2002). The paper does not, for instance, address aspects of Bayesian reasoning having to do with the alternative logical and subjectivist stances on Bayesianism, nor does it examine adherence to the dynamic coherence criterion known as the conditioning principle (for an overview of these other issues, see Baratgin and Politzer, 2006). Rather, the aspects addressed here pertain to static coherence criteria reflecting the normative view that probability is additive (Kolmogorov, 1950). Finally, although my focus is on the aforementioned aspects of Bayesianism, I neither presume nor wish to suggest that Bayesian approaches are the only viable normative frameworks for reaching probabilistic inferences under conditions of uncertainty (e.g., Lewis, 1976; Thagard, 1989; Douven and Schupbach, 2015). Indeed, as few others have noted (e.g., see Walliser and Zwirn, 2002; Baratgin and Politzer, 2006, 2010), Bayesian revision is normative in a restricted set of problem representations known as *focusing* cases—namely, cases where the original set of possible worlds is preserved rather than transformed over time. This is the type of problem studied in the present research, where only two categories exist and new information cannot invalidate either category. However, in many other cases (e.g., see Baratgin, 2009; Cozic, 2011) new information may transform the set of categories (or hypotheses) being considered. In such *updating* cases, Lewis's (1976) imaging rule provides a normative solution for probability redistribution.

For our purposes, let Ω represent an event space comprised of elementary events, *w_i_*, that is partitioned into a non-empty, closed family of subsets A. The focus in this paper is specifically on subset families that exhibit binary complementarity; namely, in which {*A*, *B*} ∈ *A*, *A* ∩ *B* = ∅ (i.e., *A* and *B* are mutually exclusive), A ≡ *A* ∪ *B* (i.e., *A* and *B* exhaustively partition A). Indeed, since *A* ⇔ ¬*B* (and likewise *B* ⇔ ¬*A*), let us use ¬*A* instead of *B* to remind ourselves that the two subsets are binary complements. For our purposes, let *H_A_* and *H*_¬*A*_ represent mutually exclusive and exhaustive hypotheses about the category membership of a focal elementary event, *w*, which in subsequent examples given in this paper is a person whose category membership is unknown. Thus, *H_A_* and *H*_¬*A*_ stand for the propositions that *w* ∈ *A* and *w* ∈ ¬*A*, respectively. In the Bayesian context, the probabilities assigned to these complementary hypotheses may be revised in light of new evidence or data, *D*. These “posterior” probabilities (see Mandel, 2014a, for an explanation of the scare quotes), *P*(*H_A_*|*D*) and *P*(*H*_¬*A*_|*D*), are the focus of most studies of Bayesian judgment, as they are in this paper.

Given the preceding definitions, the additivity principle for binary complements states that *P*(*H_A_*|*D* ∪ *H*_¬*A*_|*D*) = *P*(*H_A_*|*D*) + *P*(*H*_¬*A*_|*D*), where *P* stands for probability, a non-negative real number in the [0, 1] interval. Let *T* = *P*(*H_A_*|*D*) + *P*(*H*_¬*A*_|*D*). The complementarity constraint states that *T* = 1. In this paper, I break with the majority of papers that have followed Tversky and Koehler (1994) by calling normative violations in which *T* < 1 superadditive and violations in which *T* > 1 subadditive—terms which appear to mean precisely the opposite of what they are intended to convey. Instead, following Baratgin and Noveck (2000), I refer to cases where *T* < 1 as *subadditive* and to cases where *T* > 1 as *superadditive*. This properly places the emphasis on the additivity of the binary complements relative to unity rather than the other way around, and it is likely to be intuitive to readers outside this specific niche.

With some exceptions (e.g., Wallsten et al., 1993; Rottenstreich and Tversky, 1997; Juslin et al., 2003; see Mandel, 2005, for an explanation of differences obtained across studies), most studies have shown that people assign subadditive probabilities to binary complements (Macchi et al., 1999; Baratgin and Noveck, 2000; Windschitl et al., 2003, Experiment 4; Sloman et al., 2004; Mandel, 2005; Williams and Mandel, 2007; Mandel, 2008, Experiments 5 and 6). Additivity violations have also been shown to be systematic, following the non-normative tendency to judge *P*(*H_A_*|*D*) and *P*(*H*_¬*A*_|*D*) on the basis of their inverse probabilities, *P*(*D*|*H_A_*) and *P*(*D*|*H*_¬*A*_), respectively (Villejoubert and Mandel, 2002). This tendency has been variably called the Fisherian algorithm (Gigerenzer and Hoffrage, 1995), the confusion hypothesis (Macchi, 1995), the conversion error (Wolfe, 1995), and the inverse fallacy (Koehler, 1996). Thus, if we let *T*′ = *P*(*D*|*H_A_*) and *P*(*D*|*H*_¬*A*_), what Villejoubert and Mandel (2002) found was that subjects' *T*-values tracked the objective *T*′ values such that they were subadditive when *T*′ < 1 and superadditive when *T*′ > 1.

The second coherence constraint of interest in this paper is Bayes theorem, which is a corollary of the rule of compound probabilities, *P*(*H_A_* ∩ *D*) = *P*(*D*|*H_A_*)*P*(*H_A_*) = *P*(*H_A_*|*D*)*P*(*D*). Bayes theorem can be expressed in various ways. The most common format discussed in the literature on Bayesian reasoning performance is Bayes identity, which in general form may be expressed,

(1) $$P\left(H_{i}|D\right)=\frac{P\left(H_{i}\right)P(D|H_{i})}{P\left(D\right)}=\frac{P\left(H_{i}\right)P(D|H_{i})}{\sum_{i}P\left(H_{i}\right)P(D|H_{i})}.$$

In the case of binary complements, using the terms defined earlier, we can express Bayes identity as

(2) $$\begin{array}{l} P\left(H_{A}\text{​}|D\right)=\frac{P\left(H_{A}\right)P(D|H_{A})}{P\left(D\right)}\\ \;=\frac{P\left(H_{A}\right)P(D|H_{A})}{P\left(H_{A}\right)P\left(D\text{​}|H_{A}\right)+P\left(H_{\neg A}\right)P(D|H_{\neg A})}. \end{array}$$

However, as the rule of compound probability makes clear, Bayes theorem can also be expressed,

(3) $$P\left(H_{A}\text{​​​}|\text{​​ }D\right)=\frac{P(H_{A}\cap D)}{P\left(D\right)}=\frac{P(H_{A}\cap D)}{P\left(H_{A}\cap D\right)+P(H_{\neg A}\cap D)}.$$

When people are asked to judge *P*(*H_A_*|*D*) on the basis of information sources such as *P*(*H_A_*)—the base rate—and *P*(*D*|*H_A_*) and *P*(*D*|*H*_¬*A*_)—sometimes referred to as “diagnostic” probabilities, only a minority cohere in their judgments with Bayes theorem (e.g., Kahneman and Tversky, 1972, 1973; Lyon and Slovic, 1976; Casscells et al., 1978; Villejoubert and Mandel, 2002). For example, consider the following problem:

The probability of breast cancer is 1% for a woman at age 40 who participates in routine screening. If a woman has breast cancer, the probability is 80% that she will get a positive mammography. If a woman does not have breast cancer, the probability is 9.6% that she will also get a positive mammography. A woman in this age group had a positive mammography in a routine screening. What is the probability that she actually has breast cancer?

Using Bayes theorem, the probability that the woman has breast cancer given her test result is nearly 8%, yet Eddy (1982) found that 95 out of 100 physicians presented with the problem roughly an order of magnitude higher and similar results with physician or medical counselor samples have been found in other studies (Gigerenzer et al., 1998; Hoffrage and Gigerenzer, 1998; Garcia-Retamero and Hoffrage, 2013).

A ubiquitous explanation for the well-documented divergence between people's probability judgments and those computed on the basis of Bayes theorem is that people neglect, or at least underweight, base-rate information (Kahneman and Tversky, 1972, 1973; Lyon and Slovic, 1976; Bar-Hillel, 1980). However, without undermining the claim that base-rates are often underutilized, there is also reason to believe that the divergences reported may be due to the inverse fallacy discussed earlier (Eddy, 1982; Hamm, 1993; Koehler, 1996). For example, Villejoubert and Mandel (2002) kept base rates for two mutually exclusive and exhaustive categories equiprobable and invariant across a set of Bayesian reasoning problems. They found that most subjects judged probabilities in violation of Bayes theorem even though the possibility of base-rate underutilization was eliminated in their experiment. Moreover, the direction and magnitude of the mean difference between subjects' judgments and the Bayesian values tracked the value of the inverse probabilities, just as additivity violations had tracked the sum of the inverse probabilities^1^. As well, information search in Bayesian tasks focuses significantly more on the inverse probability of a focal hypothesis (*P*(*D*|*H_A_*)) than on either the contrapositive conditional probability (*P*(*D*|*H*_¬*A*_)) or the base-rate (*P*(*H_A_*)), and the more subjects focused on the inverse probability, the less they focused on the base rate (Wolfe, 1995). Thus, base-rate neglect may be due in part to the inverse fallacy. Finally, even in cases where base-rate neglect has been invoked as an explanation of non-conformity with Bayes theorem, such as Eddy (1982) results for the mammography problem described earlier, the inverse fallacy better accounts for the aggregate findings (Mandel, 2014a).

### Improving the coherence of probability judgments

The literature reviewed earlier shows that people often do not conform to two important coherence constraints on probability judgment when given statistical information as input to their judgment process: they systematically deviate from both the complementarity constraint and Bayes theorem. These manifestations of incoherence are particularly troubling when made by professionals whose judgments may, in turn, provide input to consequential decision-making. Much attention, as already noted, has been devoted to normative violations of probability judgment committed by medical professionals.

Another group of experts who make probabilistic judgments are intelligence analysts. Intelligence analysis plays a vital role in national and international security, serving as key sources of information for a wide range of decision-makers including state leaders, policy makers, and military commanders. Despite the importance of intelligence analysis—and the centrality of probabilistic judgment in intelligence products (Kent, 1964; Zlotnick, 1972; Friedman and Zeckhauser, 2012), there are few behavioral studies of analytical judgment quality (Pool, 2010). Probabilistic assessments underlie virtually all forecasts made by intelligence agencies. Moreover, intelligence analysts, managers, and trainers acknowledge that the predictive function of intelligence is roughly as important as the narrative descriptive function (Adams et al., 2012). Although one study has found that strategic intelligence forecasts showed good discrimination and calibration (Mandel and Barnes, 2014), the extent to which analytical judgments are coherent has not been addressed in an intelligence analyst sample. Such research is needed because intelligence analysts must often revise their hypotheses and beliefs based on missing and uncertain evidence.

Nevertheless, few, if any, analysts receive training in probabilistic belief revision. More commonly, analysts receive brief training lessons that highlight the “mindsets and biases” to which all humans are prone. In such training, analysts are taught, for instance, to “beware of overconfidence” and to “avoid confirmation bias,” but they are not routinely taught how to assess their own or others' coherence or accuracy. Few of the structured analytic techniques that analysts may use to support their assessments have been scientifically tested (Pool, 2010). Most are based on what made sense to their developers, most of whom do not have backgrounds in behavioral science. Moreover, members of the intelligence community have identified the need for evidence-based research on analytical processes that support effectiveness as a priority (Adams et al., 2012). One aim of the present research was to examine the extent to which intelligence analysts' probability judgments conform to the complementarity constraint and Bayes theorem in statistical integration tasks like the mammography problem. And a second aim was to test whether brief instruction in information structuring would have a positive effect on the quality of intelligence analysts' probability judgments. In that regard, the present research represents a rare test of the effectiveness of instruction that could be used to improve intelligence analysts' probabilistic reasoning skill.

The present research leverages recent developments in improving Bayesian reasoning. It is well established that a greater proportion of subjects in Bayesian reasoning studies provide Bayesian answers or describe a Bayesian computational process when the information provided to them is expressed in terms of natural frequencies (Gigerenzer and Hoffrage, 1995; Cosmides and Tooby, 1996; also see Kleiter, 1994). To express in natural frequencies information such as that given in the mammography problem, one would begin with a hypothetical reference class that could be easily broken down into subsamples. For instance, one might start with 1000 women aged 40 who participate in routine screening. The 1% base-rate would then be represented by subsets of 10 women who have breast cancer (*H_A_*) and 990 who do not (*H*_¬*A*_). The former subset is further decomposed into true-positive (*H_A_* ∩ *D*_+_, where *D*_+_ stands for the positive-test result) and false-negative (*H_A_* ∩ *D*_−_, where *D*_−_ stands for the negative-test result that was not obtained) subsets (8 and 2 cases, respectively), and the latter is likewise decomposed into true-negative (*H*_¬*A*_ ∩ *D*_−_) and false-positive (*H*_¬*A*_ ∩ *D*_+_) subsets (895 and 95 cases, respectively). When the information is represented as such, it is easier to calculate the “short form” of Bayes theorem shown in Equation 3. The numerator of this equation is already identified (*f*(*H_A_* ∩ *D*_+_) = 8) and the denominator simply involves adding the two subsets containing *D*_+_ (i.e., 8 + 95 = 103). Even without dividing, one might appreciate that the value 8/103 is slightly less than 8%.

Although the finding that restructuring of statistical information, such as that given in the mammography problem, into the natural frequency format just described yields better correspondence to Bayes theorem, the bases for the effect are the subject of much debate. Given that the present research does not focus on that “why” question, but rather uses the descriptive findings to explore whether Bayesian reasoning may be improved through instruction, I merely note that it is important to separate the descriptive findings from the theoretical accounts of them that have been proposed. As well, most adaptationists (e.g., see Gigerenzer and Hoffrage, 2007) and dual-systems theorists (Barbey and Sloman, 2007) do not strongly disagree that the beneficial effect of natural frequency formats derive from a combination of factors, including clarifying nested set structure of the relevant statistical data, improve the compatibility between evidence and queries, and reduce the computational complexity of task at hand (Mandel, 2007; Ayal and Beyth-Marom, 2014). More importantly, for the present purposes, most researchers agree that natural frequency presentations of statistical information in Bayesian reasoning tasks tend to facilitate Bayesian reasoning and improve Bayesian judgment.

The use of natural frequencies to convey probabilistic evidence is further augmented by the use of visual representations that reinforce the nested-set structure of diagnostic and base-rate evidence (Cosmides and Tooby, 1996). Indeed, visual representations can facilitate Bayesian reasoning by clarifying nested-set relations even when natural frequencies are not explicitly encoded in the representations (Sloman et al., 2003; Sirota et al., 2015). Such representations can also clarify the logical relations and the structure of arguments in support of alternative normative views on belief revision tasks (Mandel, 2014b). However, in at least some studies, visual representations that encode natural frequency information directly through icons or numerical values have been shown to be more effective than visualizations that clarify set structure but do not explicitly encode the frequency data, such as Euler diagrams (Sedlmeier, 1999, chapter 6; Brase, 2008, 2014). Although not all studies have shown such an advantage (e.g., Sirota et al., 2015), no study has reported the opposite effect; namely, better performance with nested-set representations that do not include explicit frequency encoding than with nested-set representations that do include such coding.

The use of visual representations of natural frequencies has also been shown to be an effective instructional method for improving compliance with Bayes theorem. Sedlmeier and Gigerenzer (2001; see also Sedlmeier, 1999) found that a single 1–2 h session of practice-based instruction in Bayesian reasoning facilitated performance on Bayesian judgment tasks. The performance boost immediately after instruction was large regardless of whether the instruction used rule-based training in the application of Bayes theorem or whether it used a natural sampling representation such as a frequency grid or frequency tree. The long-term effect of instruction, however, showed a clear advantage for instruction that relied on a natural sampling representation of the information provided in a given problem. In three experiments, on average, subjects who received such instruction performed as well at the longest-term test phase (i.e., 5 weeks in two experiments and 3 months in another experiment) as they did in the immediate test phase. In contrast, rule-based instruction showed substantial decrements by the last test phases in all experiments.^2^ The instructional benefit of frequency-based visual representations on Bayesian reasoning has been confirmed in other studies as well (Kurzenhäuser and Hoffrage, 2002; Ruscio, 2003; McCloy et al., 2007).

The present research examined the effect of instruction in information structuring on adherence to the complementarity constraint and Bayes theorem in a sample of intelligence analysts who were undergoing military intelligence training. Unlike earlier studies of instruction effects on Bayesian judgment (e.g., Sedlmeier and Gigerenzer, 2001; McCloy et al., 2007; Sirota et al., 2015), the aim of this research was not to compare different modes of instruction. Rather, the effect of a single instructional mode using a natural sampling approach with natural-frequency-tree diagrams was examined, given that this mode has already been shown to yield stable long-term improvement in conditional probability judgment. Unlike earlier research on instruction, however, this research used a pre-post design to assess the effect of instruction on complementarity constraint violations and deviations from Bayes theorem. The vast majority of studies of Bayesian reasoning have used problems with binary outcome categories corresponding to *H_A_* and *H*_¬*A*_ but have only queried subjects about one of the two hypotheses, *H_A_*. Thus, they were unable to examine the effect of Bayesian instruction on the additivity of subjects' judgments.

Moreover, the study was designed so that predictions regarding the direction of error could be made on the basis of the inverse fallacy, which, as noted earlier, has successfully accounted for both additivity violations and deviations from Bayes theorem (Villejoubert and Mandel, 2002). Specifically, assuming that the grand mean of *T* across subjects, hypotheses, and test items is additive, it was predicted that *T* < 1 if *T*′ < 1 and that *T* > 1 if *T*′ > 1. Naturally, if there were to be an overall bias toward a form of nonadditivity, the predictions would be relaxed, taking the form of the mean difference prediction *T*|(*T*′ < 1) < *T*|(*T*′ > 1). That is, a general bias in additivity would negate the predicted reflection around additivity. Given that most studies of adherence to the complementarity constraint have reported subadditivity, this form of nonadditivity is the likelier candidate. Indeed, Williams and Mandel (2007) found subadditivity for conditional probability judgments of binary complements. Although Villejoubert and Mandel (2002) did not report whether there was an overall bias in *T*, it is evident by averaging the mean *T*-values in the last column of **Table 2** in that paper that the grand mean (where the simple means were elicited within subjects) is equal to 0.916, a value that reflects subadditivity. Given that the numerical characteristics of the test items used in the present research were drawn from Villejoubert and Mandel (2002), there is good reason to expect an overall bias toward subadditivity.

Finally, an aim of the research was to examine the coherence between subjects' probability judgments and their binary forced choice of the target's category membership. Presumably, subjects would choose the category to which they assigned a higher probability. However, studies of Bayesian judgments have not asked subjects to make a discrete choice in addition to making their probability judgments. Thus, it is of interest to verify whether, in fact, subjects do invariably choose in accordance with the higher assigned probability. And, to the extent that they do not, it is of interest to examine whether instruction might attenuate this form of incoherence. Since judgments are often a precursor to decisions and actions, this is a question that is of more that academic interest.

## Materials and methods

### Subjects

Forty-three intelligence analyst trainees participated in the research during regular course time at the Canadian Forces School for Military Intelligence at Canadian Forces Base Kingston in Kingston Ontario, Canada. Twelve trainees were from a senior analysts' course, 16 were from an intermediate, basic intelligence officers' course and 15 were from a junior course. The entry requirements were an undergraduate degree for the intermediate course and completion of Grade 10 high school for the junior course. Trainees in the senior course had to have successfully completed the intermediate course. Demographic information was not recorded. However, over 90% of subjects were male. Subjects were informed that their participation was voluntary and that they would not be remunerated for their time. No student refused to participate.

### Procedure

Subjects were introduced to the study in class by being told that intelligence analysts are routinely called upon to make assessments under conditions of uncertainty, where the information they receive may be probabilistic in nature. Subjects were further told that analysts must often revise their beliefs about hypotheses or events on the basis of new, but once again, uncertain information. After this preliminary statement, subjects were informed that they had the opportunity to participate in research aimed at improving their judgment abilities. After consenting to participate, subjects were given a pre-instruction booklet that contained eight probability judgment problems, described in detail below. Participants worked on the problems individually at their desks. The task was not strictly timed. However, subjects were told that they would have approximately 15 min to complete the task. All subjects completed the task in the allotted time. An anonymous subject code was generated by the subject and written on the pre-instruction booklet before it was returned to the experimenter so that it could be matched to the post-instruction booklet.

After returning the pre-instruction booklets, the experimenter told subjects that they would now be given a brief tutorial on how to accurately integrate different sources of probabilistic information to arrive at their own probabilistic assessments of different hypotheses that one might wish to test. The first run of this experiment was conducted on the senior course and the tutorial included a series of medical diagnosis examples. The second and third runs in the other courses used an alternative version of the tutorial, which was deemed by the senior instructor at the Canadian Forces School for Military Intelligence to be more relevant to the intelligence and security context, and which focused on detecting whether a human target was an insurgent. The two versions, however, had the same structure, length, and relevant content, differing only in terms of the domain of examples (i.e., medical diagnosis vs. intelligence target detection). Both versions of the full tutorial are presented in the Supplementary Materials.

The tutorial began with an example that presents the base-rate of a focal hypothesis, *P*(*H_A_*), and diagnostic probabilities, *P*(*D*_+_|*H_A_*) and *P*(*D*_+_|*H*_¬*A*_), where *D*_+_ stands for data indicating a positive result on a diagnostic test. Subjects were asked how they might use that information to assess the conditional probability, P(*H_A_*|*D*_+_)—namely, the probability that the focal hypothesis was true given the data indicating a positive test result.

After being presented with the initial assessment task, subjects were asked to think about how they would go about making the assessment and to record their assessment. Next, the experimenter showed subjects how they could systematically work through the problem. Slides 3–5 in the tutorials were designed to show subjects how they could represent the information given to them as a natural-sampling-tree diagram. As each slide was presented, the experimenter read the textual content and pointed to the appropriate part of the diagram. Subjects were able to see the slides on a large projection screen located at the front of the classroom as well as on personal computer screens located directly in front of them on their desk spaces. On Slides 6–7, the experimenter worked through the solution, showing subjects how the information represented in the diagram could be arranged to answer the relevant question. The tutorial advises trainees to first identify the relevant set of cases that correspond to the condition, *D*_+_, specified in the conditional probability, *P*(*H_A_*|*D*_+_). Then, trainees are directed to identify the subset of those cases that conforms to the hypothesis—namely, *f*(*H_A_* ∩ *D*_+_). The corresponding diagrams made these points salient by color-coding the relevant sets of cases. The solution shown on Slide 6 represented those color-coded sets as an equation corresponding to the short form of Bayes theorem (Equation 3).

After being presented with the solution, subjects were asked to reflect on how it compared to their initial assessment (see Slide 7). Although this comparison was for pedagogical purposes, it is worth noting that many subjects commented that their estimates deviated from the correct value, and some confessed to not knowing how to integrate the information supplied (reinforcing Juslin, 2015, claim that while estimation may be very good, integration often falters).

After answering any questions subjects may have had, the experimenter moved onto the second example, which used the same cover story but asked subjects to imagine that the test result had been negative (*D*_−_) instead of positive. Subjects were asked to consider how they would assess the probability that the hypothesis was true given the negative test result, *P*(*H_A_*|*D*_−_). After subjects gave their initial assessment, the experimenter worked through the problem in the same way as before, after which subjects compared their answers to the correct solution (see Slides 10–14).

**Figure 1:**
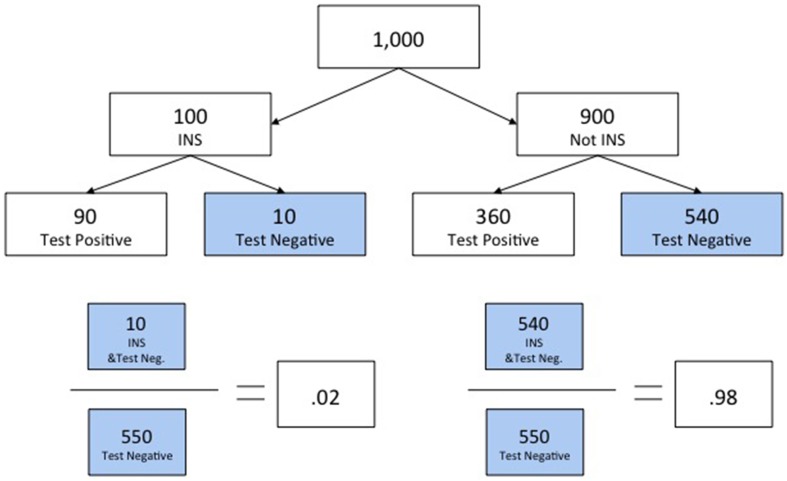
**Example from tutorial showing use of a natural sampling tree and providing solutions for assessments of alternative hypotheses defined by mutually exclusive and exhaustive subsets**.

The third example served to further illustrate that the approach taught could be used to answer other related questions, including questions framed in complementary ways (see Slide 15). Thus, whereas the second example asked subjects to assess *P*(*H_A_*|*D*_−_), the third asked them to assess the probability of the alternative hypothesis given the same negative test result, *P*(*H*_¬*A*_|*D*_−_). Once again, the solution was presented using a natural-sampling-tree diagram (see Slides 16–17). However, subjects' attention was also drawn to the fact that the answers to the two last problems summed to 100%, and they were informed that this was no coincidence. Figure 1 shows the natural-sampling-tree diagram with solutions to *P*(*H_A_*|*D*_−_) on the left and *P*(*H*_¬*A*_|*D*_−_) on the right for the intelligence version of the tutorial.

On the next slide (Slide 18), the implicit lesson about the complementarity constraint just conveyed was made explicit. Subjects were introduced to the additivity principle and told that violations of additivity represented a form of incoherence in probability assessment. The tutorial then concluded with a summary of the following key points (see Slides 19–22): first, try to visually represent the information provided, such as in the natural-sampling-tree diagrams used in the tutorial; second, in preparation for information integration, think about the probability being assessed as a ratio and identify the relevant subsets that comprise the numerator and denominator, starting with the denominator because the numerator is always a subset of the denominator; and, finally, do the arithmetic required to produce the estimate.

After answering any questions subjects may have had, the experimenter administered the post-instruction booklet to subjects, which had an alternative set of problems much like the pre-training set (detailed in subsection Judgment Tasks). Once again, subjects were given approximately 15 min to complete the set of problems and they completed the task in the allotted time. When the booklets were returned, subjects were thanked, orally debriefed, and the experiment concluded.

#### Judgment tasks

The primary judgment task assigned to subjects before and after instruction was adapted from that used by Villejoubert and Mandel (2002). The pre- and post-instruction booklets are included in the Supplementary Materials.

To summarize the task, subjects were asked to imagine that they were contestants on a game show who would be asked a series of skill-testing questions. They were to meet eight “mystery people” and, for each one, they would learn, following a query from the game-show host to the mystery person, whether a particular attribute (e.g., being a smoker) was present (*D*_+_) or absent (*D*_−_) in the individual. Half of the mystery people possessed the relevant attribute and the other half did not.

Subjects' task was to probabilistically assess the mystery person's group membership. Each person either belonged to Group A or Group B. For continuity with the prior discussion, let *H_A_* stand for the hypothesis that the target person is a member of Group A and let *H*_¬*A*_ stand for the mutually exclusive, alternative hypothesis that the target person is a member of Group B. Subjects were informed that the overall population from which the sample of eight were said to be drawn was evenly divided and, thus, *P*(*H_A_*) = *P*(*H*_¬*A*_) = 0.5. For each of the eight “encounters,” subjects also learned the diagnostic probabilities of the attribute, *P*(*D*_+_|H*_A_*) and *P*(*D*_+_|*H*_¬*A*_). Subjects were asked to estimate the probability that the target person was a member of Group A and then to estimate the probability that the person was a member of Group B on a “percentage chance” scale ranging from 0 (*absolutely no chance at all*) to 100 (*absolutely certain*) by writing a numerical value in a space provided. After giving their estimates, they were asked to make a binary choice regarding whether they thought the relevant mystery person was a member of Group A or Group B by circling one of the two options.

The diagnostic probabilities for the eight attributes (one per mystery person) are summarized in Table 1. Note that the pre- and post-instruction booklets had the same stimulus characteristics but the problems were varied by altering problem order and the attribute labels associated with each information configuration. For example, as Column 1 in Table 1 shows, the Bayesian probabilities for the encounter with mystery person 5 in the pre-instruction booklet are identical to those for the encounter with mystery person two in the post-instruction booklet. Thus, task difficulty was precisely matched between pre- and post-instruction testing sessions.

**Table 1:** **Summary of stimulus characteristics in judgment task**.

**Task no. (pre, post)**	***D***	***P*(*D*_+_|*H_A_*)**	***P*(*D*_+_|*H*_¬*A*_)**	***P*(*D*|*H_A_*)**	***P*(*D*|*H*_¬*A*_)**	***T****'*	***P*(*H_A_*|*D*)**	***P*(*H*_¬*A*_|*D*)**
5, 2	Present	0.42	0.02	0.42	0.02	0.44	0.95	0.05
6, 1	Absent	0.58	0.98	0.42	0.02	0.44	0.95	0.05
8, 3	Absent	0.40	0.80	0.60	0.20	0.80	0.75	0.25
7, 4	Present	0.60	0.20	0.60	0.20	0.80	0.75	0.25
3, 8	Present	0.80	0.40	0.80	0.40	1.20	0.67	0.33
4, 7	Absent	0.20	0.60	0.80	0.40	1.20	0.67	0.33
1, 6	Present	0.98	0.58	0.98	0.58	1.56	0.63	0.37
2, 5	Absent	0.02	0.42	0.98	0.58	1.56	0.63	0.37

*D_+_, target has attribute; D, the result for the target (either has or doesn't have attribute); H_A_, hypothesis that target belongs to Group A; H_¬A_, hypothesis that target belongs to Group B. T′ = P(D|H_A_) + P(D|H_¬A_)*.

### Design

The stimulus characteristics shown in Table 1 take the form of a 2 (Feature: present, absent) × 2 (Expected Error Direction: subadditive, superadditive) × 2 (Expected Error Magnitude: smaller, larger) within-subjects factorial design. The values of the first factor are shown in Column 2 of Table 1. The values of the second factor are encoded in column 7, where the values 0.44 and 0.80 indicate that subadditive judgments are expected if subjects commit the inverse fallacy and where the values 1.20 and 1.56 indicate that superadditive judgments are expected if subjects commit the inverse fallacy. The values 0.80 and 1.20 represent the smaller predicted errors, whereas the values 0.44 and 1.56 represent the larger predicted errors. Taking the pre-post manipulation into account, the experiment utilizes a 2 (Instruction) × 2 (Feature) × 2 (Expected Error Direction) × 2 (Expected Error Magnitude) within-subjects factorial design.

## Results

Experience, as indexed by the level of course taken (i.e., 1 = junior, 2 = intermediate, and 3 = senior), was not significantly correlated with bias (*r* = −0.07, *p* = 0.67) or absolute bias (i.e., the degree of inaccuracy irrespective of whether it represents under- or over-estimation; *r* = −0.15, *p* = 0.33). Thus, experience is not statistically controlled in subsequent analyses.

### Probability judgment

To avoid redundancy in the presentation of the results, analyses are conducted on the additivity of probability judgments for Groups A and B. The statistical analyses accompanying these analyses are, of necessity, identical in inferential characteristics, such as significance levels and effect sizes, to those focusing instead on mean bias as a measure of inaccuracy, where bias is defined as the deviation between subjects' probability judgments and the estimates based on Bayes theorem. For instance, where *T*′ = 0.44 or 1.56, a subject who invariably uses the inverse strategy would show a bias in his or her forecasts equal to |0.56|. Likewise, the subject would show an additivity violation, whereby *T* (i.e., the sum of his or her judgments for Groups A and B) would either exceed (when *T*′ = 1.56) or fall short (when *T*′ = 0.44) of unity by the same degree (i.e., 0.56).

Subjects' *T*-values were analyzed in a 2 (Instruction) × 2 (Feature) × 2 (Expected Error Direction) × 2 (Expected Error Magnitude) within-subjects factorial analysis of variance (ANOVA) model. There was a significant and large instruction effect showing that the additivity (and, by implication, mean agreement with Bayes theorem) of subjects' judgments improved from pre-instruction (*M* = 0.91, *SE* = 0.028) to post-instruction (*M* = 0.99, *SE* = 0.008) testing, *F*_(1, 42)_ = 6.82, *p* = 0.012, η^2^_*p*_ = 0.14. As the estimated marginal means show, prior to instruction, subjects' judgments, on average, were subadditive.

As predicted, the effect of instruction on additivity was moderated by the expected error direction, *F*_(1, 42)_ = 10.13, *p* = 0.003, η^2^_*p*_ = 0.19. Table 2 shows the estimated marginal mean *T*-values with 95% confidence intervals (CI). As Table 2 shows, instruction had a strong, beneficial effect on tasks in which subaddivity was predicted, *F*_(1, 42)_ = 10.27, *p* = 0.003, η^2^_*p*_ = 0.20. In that task subset, subadditivity was virtually eliminated post-instruction. In contrast, instruction had no effect when superadditivity was expected (*F* < 1). However, given that superadditivity was not found, the null effect of instruction in that context is to be expected. Rather, in that context, subjects' judgments, on average, were additive before and after instruction. No other effect in the full factorial model was significant at *p* < 0.05.

**Table 2:** **Estimated mean *T*-values by instruction and expected error direction**.

**Expected**	**Instruction**					
**Error**	**Pre**			**Post**		
**Direction**	***M***	**LB**	**UB**	***M***	**LB**	**UB**
Subadditive	0.83	0.75	0.90	0.95	0.91	0.99
Superadditive	0.99	0.91	1.07	1.02	0.99	1.05

*LB and UB = 95% CI lower and upper bounds, respectively*.

**Figure 2:**
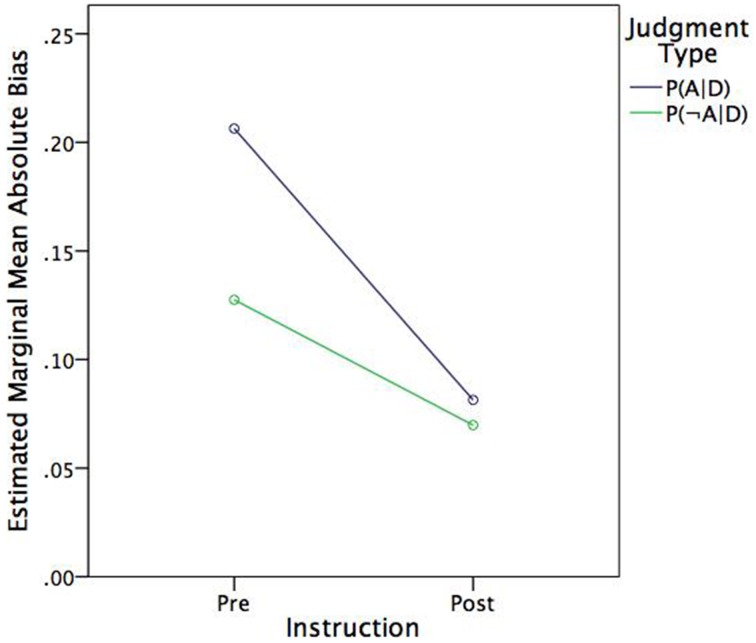
**Estimated marginal mean absolute bias by judgment type and instruction**.

The former additivity analyses showed that subjects' judgments were subadditive, which implies that, on average, they underestimated the normative estimates. As Table 1 shows, *P*(*D*|*H_A_*) > *P*(*D*|*H*_¬*A*_) and, likewise, *P*(*H_A_*|*D*) > *P*(*H*_¬*A*_|*D*). Thus, one might expect that bias expressed in absolute terms would be more pronounced for judgments of *P*(*H_A_*|*D*) than judgments of *P*(*H*_¬*A*_|*D*). To test this hypothesis, the absolute deviation between judged and normative probabilities were analyzed in a 2 (Instruction) × 2 [Judgment type: *P*(*H_A_*|*D*), *P*(*H*_¬*A*_|*D*)] within-subjects ANOVA. In fact, mean absolute bias was greater for judgments of *P*(*H_A_*|*D*) (*M* = 0.144, *SE* = 0.013) than judgments of *P*(*H*_¬*A*_|*D*) (*M* = 0.009, *SE* = 0.011), *F*_(1, 42)_ = 12.38, *p* = 0.001, η^2^_*p*_ = 0.23. As Figure 2 shows, judgment type also moderated the effect of instruction, such that there was a greater effect for judgments of *P*(*H_A_*|*D*) than judgments of *P*(*H*_¬*A*_|*D*), *F*_(1, 42)_ = *n*6.67, *p* = 0.013, η^2^_*p*_ = 0.14. In other words, instruction had a greater effect on bias reduction (i.e., improving agreement with Bayes theorem) where bias was greater to begin with.

The preceding analyses give additive analysts the benefit of the doubt. However, it is possible that some of the expressed additivity captured in this experiment is spurious. Karvetski et al. (2013) found that probability judgments of binary complements were often additive because subjects assigned values of 0.5 to *P*(*A*) and *P*(¬*A*). This pattern—known as the fifty-fifty blip (Fischhoff and Bruine de Bruin, 1999)—is likely to reflect the subjects' deep epistemic uncertainty regarding the task. Given that subjects asked to judge probabilities are seldom given a “don't know” option, they tend to express that message by responding on the midpoint of the probability scale. And when they are given a “don't know” option, fifty-fifty responses are greatly reduced (Mandel, 2005, Experiment 1b).

**Figure 3:**
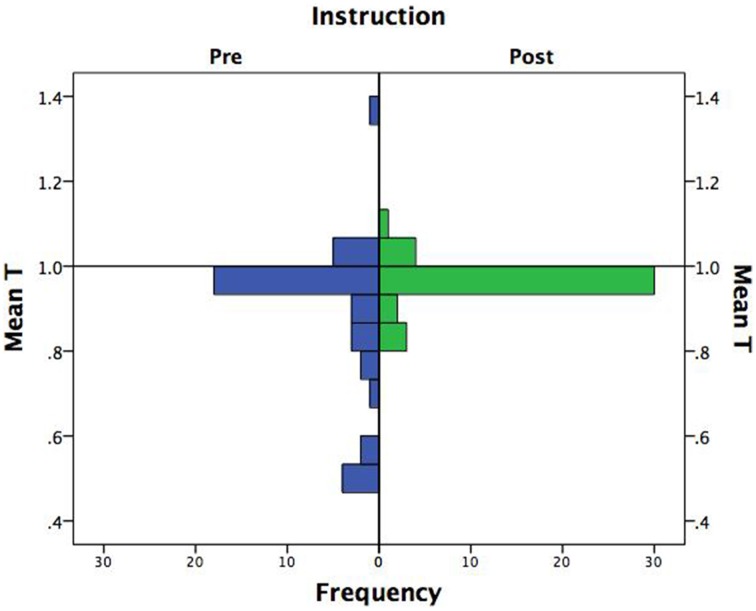
**Frequency distribution of additivity values (*T*) by instruction**.

The pre- and post-instruction test data were scanned for fifty-fifty responders. Three subjects were spuriously additive in the pre-instruction test on at least five out of the eight problems. However, no subject showed this pattern in the post-instruction test. Thus, the prior results slightly underestimate the positive instruction effect by including the spurious cases of additive judgment in the pre-instruction test phase. Deletion of the three subjects, however, had no substantial effect on the results. The main effect of instruction on subjects' *T*-values was virtually unchanged, *F*_(1, 39)_ = 6.89, *p* = 0.012, η^2^_*p*_ = 0.15; and likewise for the instruction × direction interaction effect, *F*_(1, 39)_ = 10.30, *p* = 0.003, η^2^_*p*_ = 0.21. Figure 3 shows the distribution of mean *T*-values before and after instruction with the three fifty-fifty responders excluded. It is evident that instruction was effective in improving the performance of the worst performers. In fact, the range post-instruction was less than one-third of its pre-instruction value (range = 0.26 vs. 0.88, respectively).

After removing the cases of spurious additivity, it is also of interest to compare the mean proportion of additive probability judgments before and after instruction. Instruction had a large effect on the mean proportion of additive judgments, which was greater after instruction (*M* = 0.56, *SD* = 0.42) than before instruction (*M* = 0.75, *SD* = 0.31), *t*_(39)_ = 2.86, *p* = 0.007, Cohen's *d* = 0.91. The proportion of subjects who were consistently additive across the eight problems in a test session was substantially greater after instruction (0.83) than before instruction (0.54)—a 54% increase in consistently additive responding by subjects.

### Binary choice

Although the tutorials used in this experiment did not mention choice, it was of interest to examine whether instruction may also have had a beneficial effect on the coherence of binary choices subjects made regarding the group to which the target belonged. Coherent choices are defined as those in which the subject chooses the category as the target's group to which he or she assigned the higher probability. Conversely, if the subject chooses the group to which he or she assigned the lower probability, the choice is defined as incoherent.

**Figure 4:**
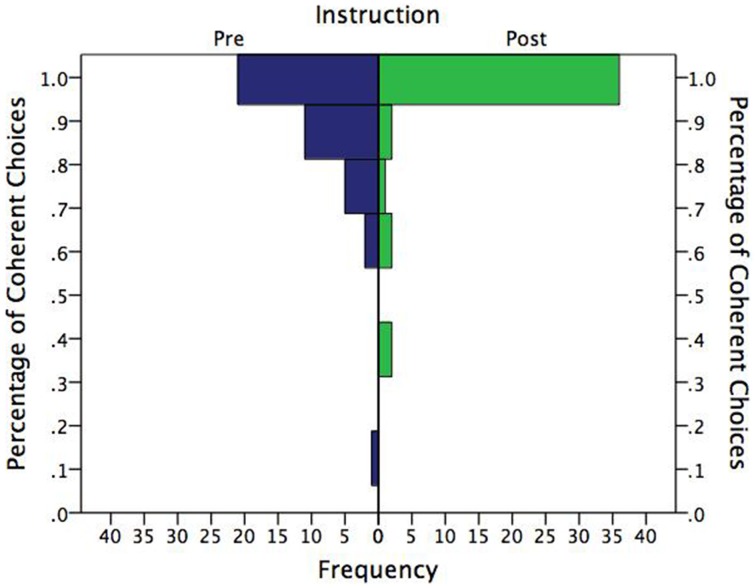
**Frequency distribution of percentage of coherent choices by instruction**.

Figure 4 shows the distribution of correct choices in percentage terms by instruction. Unsurprisingly, the distributions are highly skewed, with most subjects choosing coherently in all eight problems. What may be somewhat surprising, however, is that these distributions were not even more skewed. Clearly, the pre-instruction group showed considerable room for improvement, and improve with instruction they did. The proportion who chose coherently in all eight problems vs. those who made at least one incoherent choice was significantly greater after instruction (83%) than prior to instruction (53%), two-tailed binomial *p* = 0.002.

## Discussion

The present research adds to the body of literature showing that Bayesian reasoning can be improved through relatively brief instruction in how to structure information using natural frequency representations (Sedlmeier, 1999; Sedlmeier and Gigerenzer, 2001; Kurzenhäuser and Hoffrage, 2002; Ruscio, 2003; McCloy et al., 2007; Sirota et al., 2015). In the present experiment, brief instruction in how to represent base-rate and diagnostic probabilities as natural-frequency-tree diagrams and how to then select the relevant subsets for calculation led to a large improvement in the additivity of intelligence analysts' posterior probability judgments of binary complements. As noted earlier, this effect also reflects the degree to which those probability judgments corresponded with those given by Bayes theorem.

Consistent with the majority of previous studies that have examined violations of the complementarity constraint (Macchi et al., 1999; Baratgin and Noveck, 2000; Windschitl et al., 2003, Experiment 4; Sloman et al., 2004; Mandel, 2005; Williams and Mandel, 2007; Mandel, 2008, Experiments 5 and 6), subjects' judgments were, on average, subadditive. Nevertheless, the results also show that most subjects were consistently additive in both pre- and post- instruction test phases, with a substantial rise in that proportion after instruction. Indeed, over four-fifths of subjects answered all eight problems additively after receiving instruction. What is also striking is that over half of them did so even before receiving instruction. It is likely that these proportions were as high as they were because the binary complements were elicited in immediate succession. Prior studies (Mandel, 2005; Karvetski et al., 2013) have found that spacing binary complements apart with unrelated items or tasks reduces the likelihood of additive responses. Thus, the proportions of consistently coherent subjects obtained in this research should be interpreted as having been elicited under near ideal conditions (short of prompting subjects to make their related judgments sum to unity; e.g., see Baratgin and Noveck, 2000). It would be of value to assess the effect of instruction on additivity when the binary complements are elicited in a spaced design.

The findings also showed that the degree of subadditivity manifested across pre-instruction problem sets was consistent with use of the inverse fallacy. That is, when the inverse (i.e., diagnostic) probabilities summed to less than unity (*T*′ < 1), judgments were subadditive. In contrast, when the inverse probabilities summed to more than unity (*T*′ > 1), the pre-instruction judgments were additive—and significantly less subadditive. Nevertheless, the results of this experiment do not confirm subjects' commission of the inverse fallacy as strongly as the findings obtained by Villejoubert and Mandel (2002) because, unlike their findings which showed superadditivity when *T*′ > 1, the present findings revealed additive judgment under this condition. Simply put, exclusive reliance on the inverse fallacy in the present task would not have led to overall subadditivity.

An encouraging result was that instruction benefitted intelligence analysts' probability judgments where it was needed most. First, the effect of instruction was appropriately restricted to the subset of problems in which the inverse probabilities summed to less than unity. Under those conditions, instruction reduced additivity violations. However, for the *T*′ > 1 task subset, where subjects' judgments were additive, instruction had no effect. This null simple effect is an important result because it shows that instruction did not merely make subjects' assigned probabilities larger across the board, as some other interventions appear to have done (e.g., Williams and Mandel, 2007). The assigned probabilities only became larger where they ought to have become larger. In other words, the benefit of instruction was appropriately targeted. Second, the effect of instruction on reducing mean absolute bias was greatest for the set of judgments that yielded the greatest absolute bias in the pre-instruction test (i.e., *P*(*H_A_*|*D*)).

The benefit of instruction, as noted earlier, was also targeted in the sense that those who performed relatively poorly on the pre-instruction test, showed clear signs of improvement, as indicated by the large reduction in the range of performance post-instruction as compared to pre-instruction. This was evident in terms of both violation of the complementarity constraint and coherence of binary choices. Moreover, the few analysts who provided fifty-fifty responses prior to instruction no longer did so after instruction. These results are promising because they indicate that large improvements in probability judgment, information integration, and belief revision can be made by those who need improvement the most. Of course, the present research cannot speak to the long-term effect of instruction because the post-instruction test was administered immediately after training. However, as noted earlier, a number of studies have shown long-term beneficial effects on Bayesian judgment of instruction that has relied on the use of natural frequency representations of evidence (e.g., Sedlmeier and Gigerenzer, 2001). It would nevertheless be useful to confirm that there is a long-term benefit to judgmental coherence and also that such benefits can be derived from experts who are tasked with making judgments under conditions of uncertainty (such as intelligence analysts).

Likewise, given the encouraging results of this and other research on the use of instruction to improve aspects of Bayesian judgment, it would be of value to explore how such instruction might be further optimized by incorporating other effective learning techniques (for overviews, see Dunlosky et al., 2013; Kober, 2015). For instance, most studies of instruction effects on Bayesian reasoning, including the present research, have used a massed training and practice session. However, much experimental evidence indicates that students learn more effectively when they are given opportunities for distributed practice with large time lags between sessions (Cepeda et al., 2006; Delaney et al., 2010). While the majority of studies have demonstrated the benefits of distributed practice using factual materials that require mainly recall ability, Kapler et al. (2014) have shown that distributed practice in a simulated undergraduate classroom improves learning of higher-level reasoning that requires both recall and manipulation of information, much as Bayesian reasoning requires.

Finally, it is worth noting that the present research yielded not only a large statistical effect but also a practical effect given that the instructional method developed and tested in this research has since been adopted in some intelligence courses in Canada. Of course, it remains unclear to what extent such training will ultimately affect the quality of intelligence analysis and whether, in fact, Bayesianism is an appropriate model for belief revision in that domain (for an insightful discussion, see Zlotnick, 1972). Given that most assessments are communicated with verbal probability phrases and few assessments are based on evidence for which uncertainties are quantified, the application of aspects of Bayesianism such as Bayes theorem are currently of limited value. Nevertheless, even verbal probabilities should respect coherence principles such as additivity. It may be more difficult to verify whether “very likely that *A* will happen” and “slim chance that *A* won't happen” add up to unity, and such verification would be less direct because it would require personally translating the phrases into numbers. However, even without translation attempts, one could be reasonably confident that “almost certain that it's *A*” and “fifty-fifty that it's not *A*” are superadditive. Moreover, judgment accuracy is substantially improved by giving subjects in an opinion pool weight proportional to their adherence to the additivity principle (Karvetski et al., 2013). Forecast accuracy has also been improved by probability training that took the form of directives and rules of thumb aimed at avoiding common pitfalls, such as assigning probabilities of fifty-fifty to binary complements when forecasters are deeply unsure (Mellers et al., 2014). The instructional method developed in this research could potentially be used on its own or in combination with directive-based probability training to improve the quality of forecasting in the intelligence community and in other expert domains requiring probability judgment.

### Conflict of interest statement

The author declares that the research was conducted in the absence of any commercial or financial relationships that could be construed as a potential conflict of interest.

## Footnotes

^1^This is by necessity: if *T* < 1, then the mean bias (i.e., the mean deviation between the subject's posterior probability and the values given by Bayes theorem) must be negative, representing underestimation, by the same degree. Likewise when *T* > 1; then, mean bias must represent overestimation to the same degree.

^2^The one exception was in Study 1b of Sedlmeier and Gigerenzer (2001) where subjects were incentivized through bonuses and where rule-based and natural sampling methods yielded comparable performance.
