# Supplementary material for: Instruction in information structuring improves Bayesian judgment in intelligence analysts
Source: Front Psychol. 2015 Apr 8;6:387. doi: 10.3389/fpsyg.2015.00387 (PMC4389401; doi:10.3389/fpsyg.2015.00387)
Supplement: Supplementary file 1 [file Presentation1.PDF]

Supplementary Material

Instruction in Information Structuring Improves Bayesian Judgment in Intelligence Analysts

David R. Mandel*
Socio-Cognitive Systems Section, Defence Research and Development Canada and Department of Psychology, York University, Toronto, Ontario, Canada

***Correspondence:** David R. Mandel, Socio-Cognitive Systems Section, Defence Research and Development Canada, 1133 Sheppard Avenue West, Toronto, Ontario, M3K 2C9, Canada
david.mandel@drdc-rddc.gc.ca

1. Supplementary Data

The data file is provided in Excel format.

2. Supplementary Materials

2.1. Instructional Materials

The instructional materials used in this research are provided as PDF files.

2.1 Pre- and Post-instruction Test Measures

The pre- and post-instruction test measures are presented below, respectively.

Booklet #1 [Pre-instructional test]

Participant Code: Combine the first two letters of your mother's first name with the last four digits of your home phone number. For example, if your mother's name is Mary and your home phone number is (613) 549-2682, your participant code would be **MA2682**

Participant Code: ________________

In the next section of this study, please imagine that you are a contestant on a game show who will complete a series of skill-testing questions.

As a contestant in this game show, you will meet eight "mystery people" one person at a time. In each case, your task is to classify the mystery person into one of two categories – namely, Group A or Group B.

Each of these groups has 100 members in total. The eight mystery people that you will meet have been randomly selected from the total pool of members in Groups A and B (namely from the entire 200 people).

After a mystery person is introduced to you by their number in the sequence (namely, Mystery Person #1 to Mystery Person #8), the game-show host will ask the mystery person a question about a characteristic that he or she might possess (e.g., "Mystery Person #1, do you watch television?"). The mystery person will then answer the question – *always honestly* – by either saying YES or NO.

To assist you in your task, the host will then give you some additional information about the likelihood of that same characteristic (e.g., "watching television") among members of Group A and among members of Group B. For instance, the host might tell you that 50% of Group A watches television and 75% of Group B watches television.

Finally, the host will ask you three questions:

(1) to judge the probability that the mystery person is a member of Group A
(2) to judge the probability that the mystery person is from Group B, and
(3) to pick the group to which you think the mystery person belongs.

Now, please turn the page to begin the game by meeting Mystery Person #1.

Host: MYSTERY PERSON #1, I will now ask you a question and you will respond honestly. The question is: Do you smoke cigarettes?

Mystery Person #1: Yes, I do smoke cigarettes.

Host: Thank you! Now Contestant, to make your task easier, I will also let you know that 98% of members of Group A smoke cigarettes and 58% of members of Group B smoke cigarettes.

Host: Now Contestant, on the basis of what you know from the Mystery Person's response and the information I gave you, please answer the following three questions:

1. What is your estimate of the probability that Mystery Person #1 belongs to Group A? Please indicate your response by writing down a value from 0 to 100 where 0 stands for "*absolutely no chance at all*" and 100 stands for "*absolutely certain*".

   The probability that this individual belongs to Group A is _______.

2. What is your estimate of the probability that Mystery Person #1 belongs to Group B? Please indicate your response by writing down a value from 0 to 100 where 0 stands for "*absolutely no chance at all*" and 100 stands for "*absolutely certain*".

   The probability that this individual belongs to Group B is _______.

3. Finally, if you had to pick Group A or Group B as the group that Mystery Person #1 belongs to, which would it be? Circle one:

   [GROUP A]   [GROUP B]

Host: Thank you for your responses. Now please turn the page to meet Mystery Person #2.

Host: MYSTERY PERSON #2, I will now ask you a question and you will respond honestly. The question is: Do you eat seafood?

Mystery Person #2: No, I do not eat seafood.

Host: Thank you! Now Contestant, to make your task easier, I will also let you know that 2% of members of Group A eat seafood and 42% of members of Group B eat seafood.

Host: Now Contestant, on the basis of what you know from the Mystery Person's response and the information I gave you, please answer the following three questions:

4. What is your estimate of the probability that Mystery Person #2 belongs to Group A? Please indicate your response by writing down a value from 0 to 100 where 0 stands for "*absolutely no chance at all*" and 100 stands for "*absolutely certain*".

   The probability that this individual belongs to Group A is _______.

5. What is your estimate of the probability that Mystery Person #2 belongs to Group B? Please indicate your response by writing down a value from 0 to 100 where 0 stands for "*absolutely no chance at all*" and 100 stands for "*absolutely certain*".

   The probability that this individual belongs to Group B is _______.

6. Finally, if you had to pick Group A or Group B as the group that Mystery Person #2 belongs to, which would it be? Circle one:

   [GROUP A]   [GROUP B]

Host: Thank you for your responses. Now please turn the page to meet Mystery Person #3.

Host: MYSTERY PERSON #3, I will now ask you a question and you will respond honestly. The question is: Do you play the harmonica?

Mystery Person #3: Yes, I do play the harmonica.

Host: Thank you! Now Contestant, to make your task easier, I will also let you know that 80% of members of Group A play the harmonica and 40% of members of Group B play the harmonica.

Host: Now Contestant, on the basis of what you know from the Mystery Person's response and the information I gave you, please answer the following three questions:

7. What is your estimate of the probability that Mystery Person #3 belongs to Group A? Please indicate your response by writing down a value from 0 to 100 where 0 stands for "*absolutely no chance at all*" and 100 stands for "*absolutely certain*".

   The probability that this individual belongs to Group A is _______.

8. What is your estimate of the probability that Mystery Person #3 belongs to Group B? Please indicate your response by writing down a value from 0 to 100 where 0 stands for "*absolutely no chance at all*" and 100 stands for "*absolutely certain*".

   The probability that this individual belongs to Group B is _______.

9. Finally, if you had to pick Group A or Group B as the group that Mystery Person #3 belongs to, which would it be? Circle one:

   [GROUP A] [GROUP B]

Host: Thank you for your responses. Now please turn the page to meet Mystery Person #4.

Host: MYSTERY PERSON #4, I will now ask you a question and you will respond honestly. The question is: Do you have a flying license?

Mystery Person #4: No, I do not have a flying license.

Host: Thank you! Now Contestant, to make your task easier, I will also let you know that 20% of members of Group A have a flying license and 60% of members of Group B have a flying license.

Host: Now Contestant, on the basis of what you know from the Mystery Person's response and the information I gave you, please answer the following three questions:

10. What is your estimate of the probability that Mystery Person #4 belongs to Group A? Please indicate your response by writing down a value from 0 to 100 where 0 stands for "*absolutely no chance at all*" and 100 stands for "*absolutely certain*".

    The probability that this individual belongs to Group A is _______.

11. What is your estimate of the probability that Mystery Person #4 belongs to Group B? Please indicate your response by writing down a value from 0 to 100 where 0 stands for "*absolutely no chance at all*" and 100 stands for "*absolutely certain*".

    The probability that this individual belongs to Group B is _______.

12. Finally, if you had to pick Group A or Group B as the group that Mystery Person #4 belongs to, which would it be? Circle one:

    [GROUP A]   [GROUP B]

Host: Thank you for your responses. Now please turn the page to meet Mystery Person #5.

Host: MYSTERY PERSON #5, I will now ask you a question and you will respond honestly. The question is: Are you afraid of heights?

Mystery Person #5: Yes, I am afraid of heights.

Host: Thank you! Now Contestant, to make your task easier, I will also let you know that 42% of members of Group A are afraid of heights and 2% of members of Group B are afraid of heights.

Host: Now Contestant, on the basis of what you know from the Mystery Person's response and the information I gave you, please answer the following three questions:

13. What is your estimate of the probability that Mystery Person #5 belongs to Group A? Please indicate your response by writing down a value from 0 to 100 where 0 stands for "*absolutely no chance at all*" and 100 stands for "*absolutely certain*".

    The probability that this individual belongs to Group A is _______.

14. What is your estimate of the probability that Mystery Person #5 belongs to Group B? Please indicate your response by writing down a value from 0 to 100 where 0 stands for "*absolutely no chance at all*" and 100 stands for "*absolutely certain*".

    The probability that this individual belongs to Group B is _______.

15. Finally, if you had to pick Group A or Group B as the group that Mystery Person #5 belongs to, which would it be? Circle one:

    [GROUP A]   [GROUP B]

Host: Thank you for your responses. Now please turn the page to meet Mystery Person #6.

Host: MYSTERY PERSON #6, I will now ask you a question and you will respond honestly. The question is: Do you ride a skateboard?

Mystery Person #6: No, I do not ride a skateboard.

Host: Thank you! Now Contestant, to make your task easier, I will also let you know that 58% of members of Group A ride a skateboard and 98% of members of Group B ride a skateboard.

Host: Now Contestant, on the basis of what you know from the Mystery Person's response and the information I gave you, please answer the following three questions:

16. What is your estimate of the probability that Mystery Person #6 belongs to Group A? Please indicate your response by writing down a value from 0 to 100 where 0 stands for "*absolutely no chance at all*" and 100 stands for "*absolutely certain*".

    The probability that this individual belongs to Group A is _______.

17. What is your estimate of the probability that Mystery Person #6 belongs to Group B? Please indicate your response by writing down a value from 0 to 100 where 0 stands for "*absolutely no chance at all*" and 100 stands for "*absolutely certain*".

    The probability that this individual belongs to Group B is _______.

18. Finally, if you had to pick Group A or Group B as the group that Mystery Person #6 belongs to, which would it be? Circle one:

    [GROUP A]   [GROUP B]

Host: Thank you for your responses. Now please turn the page to meet Mystery Person #7.

Host: MYSTERY PERSON #7, I will now ask you a question and you will respond honestly. The question is: Have you ever been a firefighter?

Mystery Person #7: Yes, I have been a firefighter.

Host: Thank you! Now Contestant, to make your task easier, I will also let you know that 60% of members of Group A have been a firefighter and 20% of members of Group B have been a firefighter.

Host: Now Contestant, on the basis of what you know from the Mystery Person's response and the information I gave you, please answer the following three questions:

19. What is your estimate of the probability that Mystery Person #7 belongs to Group A? Please indicate your response by writing down a value from 0 to 100 where 0 stands for "*absolutely no chance at all*" and 100 stands for "*absolutely certain*".

    The probability that this individual belongs to Group A is _______.

20. What is your estimate of the probability that Mystery Person #7 belongs to Group B? Please indicate your response by writing down a value from 0 to 100 where 0 stands for "*absolutely no chance at all*" and 100 stands for "*absolutely certain*".

    The probability that this individual belongs to Group B is _______.

21. Finally, if you had to pick Group A or Group B as the group that Mystery Person #7 belongs to, which would it be? Circle one:

    [GROUP A] [GROUP B]

Host: Thank you for your responses. Now please turn the page to meet Mystery Person #8.

Host: MYSTERY PERSON #8, I will now ask you a question and you will respond honestly. The question is: Do you drink beer?

Mystery Person #8: No, I do not drink beer.

Host: Thank you! Now Contestant, to make your task easier, I will also let you know that 40% of members of Group A drink beer and 80% of members of Group B drink beer.

Host: Now Contestant, on the basis of what you know from the Mystery Person's response and the information I gave you, please answer the following three questions:

22. What is your estimate of the probability that Mystery Person #8 belongs to Group A? Please indicate your response by writing down a value from 0 to 100 where 0 stands for "*absolutely no chance at all*" and 100 stands for "*absolutely certain*".

    The probability that this individual belongs to Group A is _______.

23. What is your estimate of the probability that Mystery Person #8 belongs to Group B? Please indicate your response by writing down a value from 0 to 100 where 0 stands for "*absolutely no chance at all*" and 100 stands for "*absolutely certain*".

    The probability that this individual belongs to Group B is _______.

24. Finally, if you had to pick Group A or Group B as the group that Mystery Person #8 belongs to, which would it be? Circle one:

    [GROUP A]   [GROUP B]

THANK YOU FOR COMPLETING THE STUDY.
PLEASE LET THE EXPERIMENTER KNOW THAT YOU HAVE FINISHED.

Booklet #2 [Post-instruction test]

Participant Code: Combine the first two letters of your mother's first name with the last four digits of your home phone number. For example, if your mother's name is Mary and your home phone number is (613) 549-2682, your participant code would be **MA2682**

Participant Code: ________________

In the next section of this study, please imagine that you are a contestant on a game show who will complete a series of skill-testing questions.

As a contestant in this game show, you will meet eight "mystery people" one person at a time. In each case, your task is to classify the mystery person into one of two categories – namely, Group A or Group B.

Each of these groups has 100 members in total. The eight mystery people that you will meet have been randomly selected from the total pool of members in Groups A and B (namely from the entire 200 people).

After a mystery person is introduced to you by their number in the sequence (namely, Mystery Person #1 to Mystery Person #8), the game-show host will ask the mystery person a question about a characteristic that he or she might possess (e.g., "Mystery Person #1, do you watch television?"). The mystery person will then answer the question – *always honestly* – by either saying YES or NO.

To assist you in your task, the host will then give you some additional information about the likelihood of that same characteristic (e.g., "watching television") among members of Group A and among members of Group B. For instance, the host might tell you that 50% of Group A watches television and 75% of Group B watches television.

Finally, the host will ask you three questions:

(4) to judge the probability that the mystery person is a member of Group A
(5) to judge the probability that the mystery person is from Group B, and
(6) to pick the group to which you think the mystery person belongs.

Now, please turn the page to begin the game by meeting Mystery Person #1.

Host: MYSTERY PERSON #1, I will now ask you a question and you will respond honestly. The question is: Do you drive a car?

Mystery Person #1: No, I do not drive a car.

Host: Thank you! Now Contestant, to make your task easier, I will also let you know that 58% of members of Group A drive a car and 98% of members of Group B drive a car.

Host: Now Contestant, on the basis of what you know from the Mystery Person's response and the information I gave you, please answer the following three questions:

25. What is your estimate of the probability that Mystery Person #1 belongs to Group A? Please indicate your response by writing down a value from 0 to 100 where 0 stands for "*absolutely no chance at all*" and 100 stands for "*absolutely certain*".

    The probability that this individual belongs to Group A is _______.

26. What is your estimate of the probability that Mystery Person #1 belongs to Group B? Please indicate your response by writing down a value from 0 to 100 where 0 stands for "*absolutely no chance at all*" and 100 stands for "*absolutely certain*".

    The probability that this individual belongs to Group B is _______.

27. Finally, if you had to pick Group A or Group B as the group that Mystery Person #1 belongs to, which would it be? Circle one:

    [GROUP A]   [GROUP B]

Host: Thank you for your responses. Now please turn the page to meet Mystery Person #2.

Host: MYSTERY PERSON #2, I will now ask you a question and you will respond honestly. The question is: Have you ever travelled to Europe?

Mystery Person #2: Yes, I have travelled to Europe.

Host: Thank you! Now Contestant, to make your task easier, I will also let you know that 42% of members of Group A have travelled to Europe and 2% of members of Group B have travelled to Europe.

Host: Now Contestant, on the basis of what you know from the Mystery Person's response and the information I gave you, please answer the following three questions:

28. What is your estimate of the probability that Mystery Person #2 belongs to Group A? Please indicate your response by writing down a value from 0 to 100 where 0 stands for "*absolutely no chance at all*" and 100 stands for "*absolutely certain*".

    The probability that this individual belongs to Group A is _______.

29. What is your estimate of the probability that Mystery Person #2 belongs to Group B? Please indicate your response by writing down a value from 0 to 100 where 0 stands for "*absolutely no chance at all*" and 100 stands for "*absolutely certain*".

    The probability that this individual belongs to Group B is _______.

30. Finally, if you had to pick Group A or Group B as the group that Mystery Person #2 belongs to, which would it be? Circle one:

    [GROUP A]   [GROUP B]

Host: Thank you for your responses. Now please turn the page to meet Mystery Person #3.

Host: MYSTERY PERSON #3, I will now ask you a question and you will respond honestly. The question is: Do you enjoy watching The Simpsons?

Mystery Person #3: No, I do not enjoy watching The Simpsons.

Host: Thank you! Now Contestant, to make your task easier, I will also let you know that 40% of members of Group A enjoy watching The Simpsons and 80% of members of Group B enjoy watching The Simpsons.

Host: Now Contestant, on the basis of what you know from the Mystery Person's response and the information I gave you, please answer the following three questions:

31. What is your estimate of the probability that Mystery Person #3 belongs to Group A? Please indicate your response by writing down a value from 0 to 100 where 0 stands for "*absolutely no chance at all*" and 100 stands for "*absolutely certain*".

    The probability that this individual belongs to Group A is _______.

32. What is your estimate of the probability that Mystery Person #3 belongs to Group B? Please indicate your response by writing down a value from 0 to 100 where 0 stands for "*absolutely no chance at all*" and 100 stands for "*absolutely certain*".

    The probability that this individual belongs to Group B is _______.

33. Finally, if you had to pick Group A or Group B as the group that Mystery Person #3 belongs to, which would it be? Circle one:

    [GROUP A]   [GROUP B]

Host: Thank you for your responses. Now please turn the page to meet Mystery Person #4.

Host: MYSTERY PERSON #4, I will now ask you a question and you will respond honestly. The question is: Do you own a home?

Mystery Person #4: Yes, I own a home.

Host: Thank you! Now Contestant, to make your task easier, I will also let you know that 60% of members of Group A own a home and 20% of members of Group B own a home.

Host: Now Contestant, on the basis of what you know from the Mystery Person's response and the information I gave you, please answer the following three questions:

34. What is your estimate of the probability that Mystery Person #4 belongs to Group A? Please indicate your response by writing down a value from 0 to 100 where 0 stands for "*absolutely no chance at all*" and 100 stands for "*absolutely certain*".

    The probability that this individual belongs to Group A is _______.

35. What is your estimate of the probability that Mystery Person #4 belongs to Group B? Please indicate your response by writing down a value from 0 to 100 where 0 stands for "*absolutely no chance at all*" and 100 stands for "*absolutely certain*".

    The probability that this individual belongs to Group B is _______.

36. Finally, if you had to pick Group A or Group B as the group that Mystery Person #4 belongs to, which would it be? Circle one:

    [GROUP A] [GROUP B]

Host: Thank you for your responses. Now please turn the page to meet Mystery Person #5.

Host: MYSTERY PERSON #5, I will now ask you a question and you will respond honestly. The question is: D you like to dance?

Mystery Person #5: No, I do not like to dance.

Host: Thank you! Now Contestant, to make your task easier, I will also let you know that 2% of members of Group A like to dance and 42% of members of Group B like to dance.

Host: Now Contestant, on the basis of what you know from the Mystery Person's response and the information I gave you, please answer the following three questions:

37. What is your estimate of the probability that Mystery Person #5 belongs to Group A? Please indicate your response by writing down a value from 0 to 100 where 0 stands for "*absolutely no chance at all*" and 100 stands for "*absolutely certain*".

    The probability that this individual belongs to Group A is _______.

38. What is your estimate of the probability that Mystery Person #5 belongs to Group B? Please indicate your response by writing down a value from 0 to 100 where 0 stands for "*absolutely no chance at all*" and 100 stands for "*absolutely certain*".

    The probability that this individual belongs to Group B is _______.

39. Finally, if you had to pick Group A or Group B as the group that Mystery Person #5 belongs to, which would it be? Circle one:

    [GROUP A]   [GROUP B]

Host: Thank you for your responses. Now please turn the page to meet Mystery Person #6.

Host: MYSTERY PERSON #6, I will now ask you a question and you will respond honestly. The question is: Do you own a pet?

Mystery Person #6: Yes, I own a pet.

Host: Thank you! Now Contestant, to make your task easier, I will also let you know that 98% of members of Group A own a pet and 58% of members of Group B own a pet.

Host: Now Contestant, on the basis of what you know from the Mystery Person's response and the information I gave you, please answer the following three questions:

40. What is your estimate of the probability that Mystery Person #6 belongs to Group A? Please indicate your response by writing down a value from 0 to 100 where 0 stands for "*absolutely no chance at all*" and 100 stands for "*absolutely certain*".

    The probability that this individual belongs to Group A is _______.

41. What is your estimate of the probability that Mystery Person #6 belongs to Group B? Please indicate your response by writing down a value from 0 to 100 where 0 stands for "*absolutely no chance at all*" and 100 stands for "*absolutely certain*".

    The probability that this individual belongs to Group B is _______.

42. Finally, if you had to pick Group A or Group B as the group that Mystery Person #6 belongs to, which would it be? Circle one:

    [GROUP A]   [GROUP B]

Host: Thank you for your responses. Now please turn the page to meet Mystery Person #7.

Host: MYSTERY PERSON #7, I will now ask you a question and you will respond honestly. The question is: Have you ever voted in a federal election?

Mystery Person #7: No, I have never voted in a federal election.

Host: Thank you! Now Contestant, to make your task easier, I will also let you know that 20% of members of Group A have voted in a federal election and 60% of members of Group B have voted in a federal election.

Host: Now Contestant, on the basis of what you know from the Mystery Person's response and the information I gave you, please answer the following three questions:

43. What is your estimate of the probability that Mystery Person #7 belongs to Group A? Please indicate your response by writing down a value from 0 to 100 where 0 stands for "*absolutely no chance at all*" and 100 stands for "*absolutely certain*".

    The probability that this individual belongs to Group A is _______.

44. What is your estimate of the probability that Mystery Person #7 belongs to Group B? Please indicate your response by writing down a value from 0 to 100 where 0 stands for "*absolutely no chance at all*" and 100 stands for "*absolutely certain*".

    The probability that this individual belongs to Group B is _______.

45. Finally, if you had to pick Group A or Group B as the group that Mystery Person #7 belongs to, which would it be? Circle one:

    [GROUP A]   [GROUP B]

Host: Thank you for your responses. Now please turn the page to meet Mystery Person #8.

Host: MYSTERY PERSON #8, I will now ask you a question and you will respond honestly. The question is: Can you swim?

Mystery Person #8: Yes, I can swim.

Host: Thank you! Now Contestant, to make your task easier, I will also let you know that 80% of members of Group A can swim and 40% of members of Group B can swim.

Host: Now Contestant, on the basis of what you know from the Mystery Person's response and the information I gave you, please answer the following three questions:

46. What is your estimate of the probability that Mystery Person #8 belongs to Group A? Please indicate your response by writing down a value from 0 to 100 where 0 stands for "*absolutely no chance at all*" and 100 stands for "*absolutely certain*".

    The probability that this individual belongs to Group A is _______.

47. What is your estimate of the probability that Mystery Person #8 belongs to Group B? Please indicate your response by writing down a value from 0 to 100 where 0 stands for "*absolutely no chance at all*" and 100 stands for "*absolutely certain*".

    The probability that this individual belongs to Group B is _______.

48. Finally, if you had to pick Group A or Group B as the group that Mystery Person #8 belongs to, which would it be? Circle one:

    [GROUP A] [GROUP B]

THANK YOU FOR COMPLETING THIS STUDY.
PLEASE LET THE EXPERIMENTER KNOW THAT YOU HAVE FINISHED.
