# Supplementary material for: Instruction in information structuring improves Bayesian judgment in intelligence analysts
Source: Front Psychol. 2015 Apr 8;6:387. doi: 10.3389/fpsyg.2015.00387 (PMC4389401; doi:10.3389/fpsyg.2015.00387)
Supplement: Supplementary file 2 [file Presentation2.PDF]

# Calculating posterior probabilities from base rates and diagnostic probabilities

This tutorial will take you through a series of examples that illustrate how to correctly estimate probability from available evidence.

## Example 1: Medical Judgment

Imagine that you are a doctor and you suspect that one of your patients (John) might have Hepatitis C (Hep-C). You tested John for the illness and the test result was positive. Now, John would like to know the probability of actually having the virus given that he tested positive. Assume the following statistics are accurate: Hep-C is found in 10% of the general population, and 90% of people who have Hep-C test positive on the same test you gave to John. The research also indicates that 40% of people who do <u>not</u> have Hep-C also test positive on this test. This is because the test is not 100% accurate. It makes an incorrect diagnosis 10% of time for people who have Hep-C and 40% of time for people who do not have Hep-C.

Now, if you wanted to assess the probability that John has Hep-C given that he tested positive, how would you go about doing that? Let's begin by having you provide your initial estimate of this probability. Then we will work through a systematic way of solving this type of problem. Before advancing to the next screen, please write your estimate in percentages from 0 (no chance) to 100 (absolutely certain) in the booklet provided.

We will work through one approach that presents the solution to a query about probability as a graphically illustrated step-by-step process. First, let's start with a relatively large sample of the general population—say, 1,000 people. Then we'll divide this sample into the percentage that has Hep-C and the remaining percentage that does not have Hep-C. Based on the information given earlier, we'd expect that 100 of the 1,000 people (10%) will have Hep-C and the remaining 900 (90%) will not.

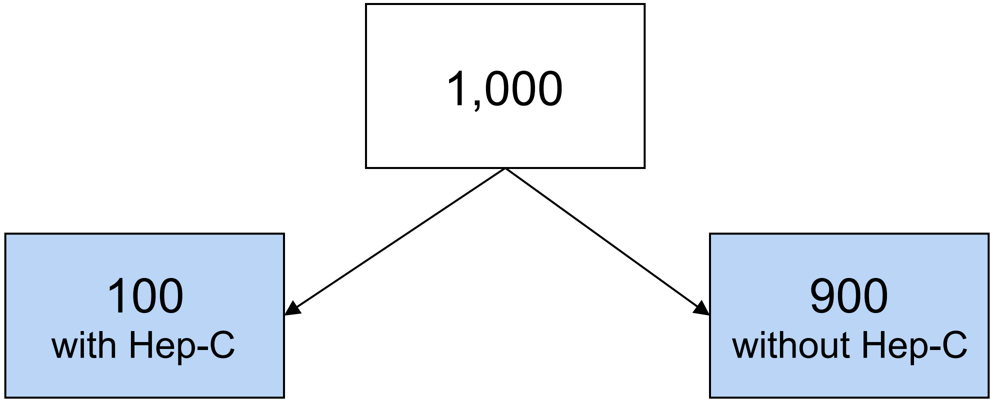

Next, we can break down each of the sub-samples into those who are likely to test positive and those who are likely to test negative. Let’s do this first for the people who are expected to have Hep-C. Based on the information reported earlier, we know that 90% of people with Hep-C test positive on the test that John received—namely, they are correctly diagnosed as having the illness. That would be 90 people in this example. The remaining 10% (10 people) do not test positive—namely, they are falsely diagnosed as not having the illness. The breakdown is shown graphically below:

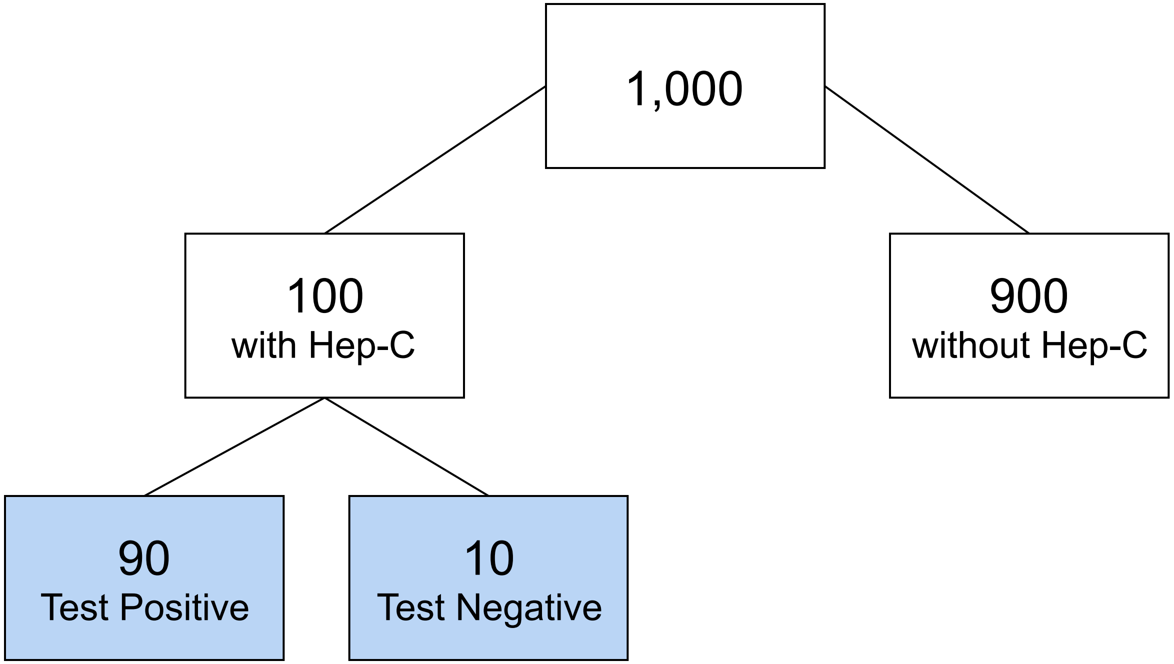

Now, we'll do the same thing for the 900 people without Hep-C. Based on the information reported earlier, we know that 40% of people without Hep-C test positive on the test that John received—namely, they are misdiagnosed as having the illness. The remaining 60% do not test positive—namely, they are correctly diagnosed as not having the illness. Since 40% of 900 is 360 and 60% of 900 is 540, we can show the breakdown as follows:

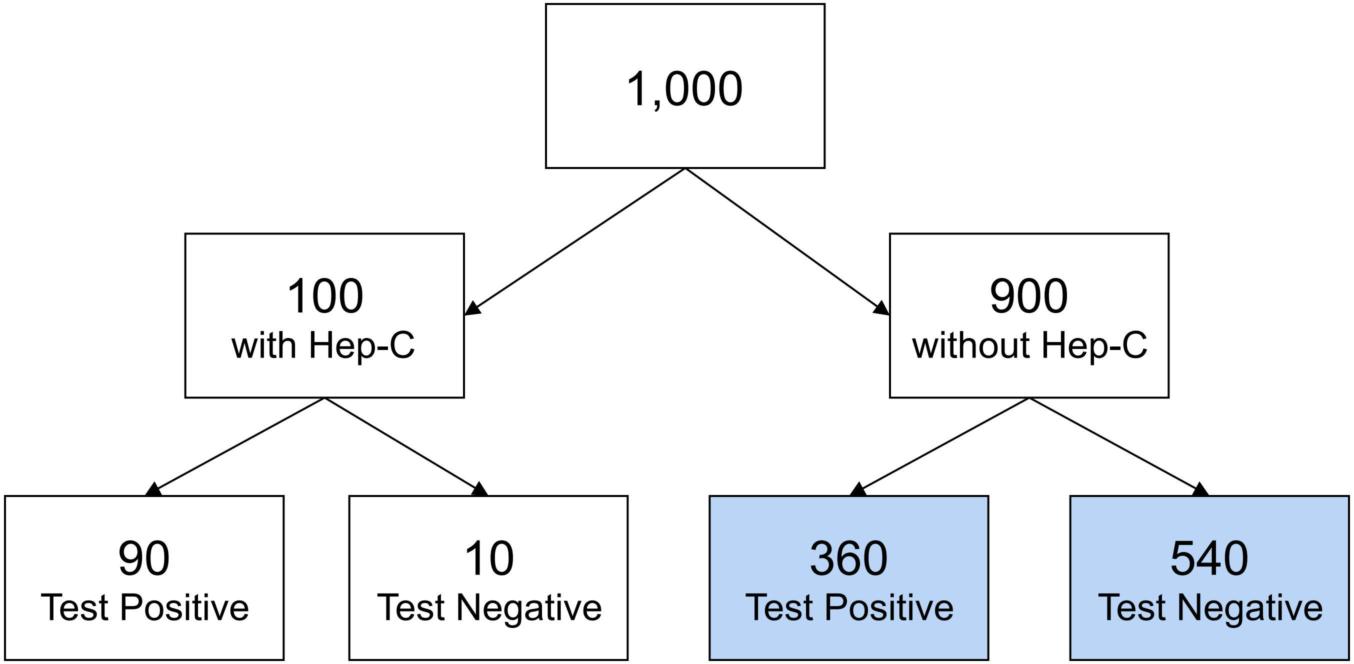

Now that we've illustrated how the probability information given at the start of the problem could be clearly visually represented, the next question is what do we do with it in order to answer the question that John originally posed—namely, *what is his probability of having Hep-C given that he tested positive*?

First, we need to determine the number of people in our sample who are expected to test positive like John. To do so, we simply add the number of all people who test positive regardless of whether they have Hep-C (90) or not (360). These values sum to 450.

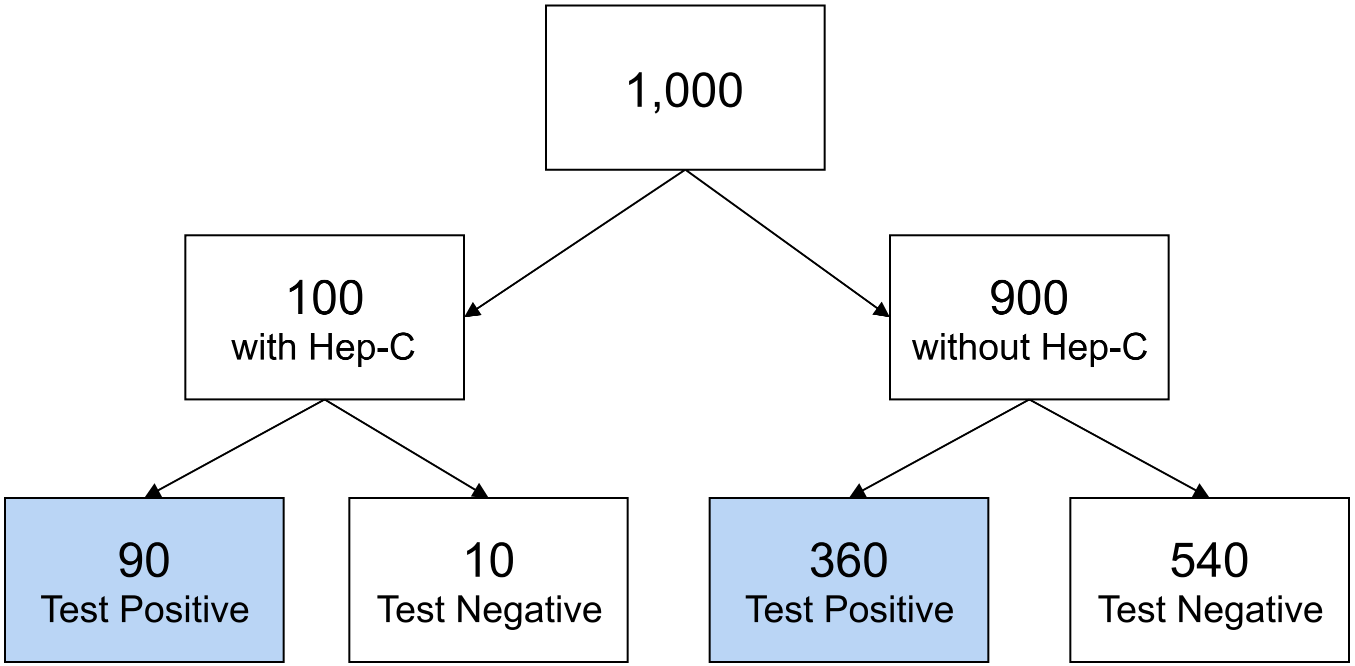

We're almost there! To figure out the probability of John having Hep-C given that he tested positive, you will want to know how many out of the 450 people who test positive also have Hep-C. That ratio will be the answer to John's question. Since we know that 90 of the 450 people who test positive also have Hep-C, we simply divide 90 over 450 to get the answer. Since 90/450 = .20, there is exactly a 20% chance that John has Hep-C given his positive test result.

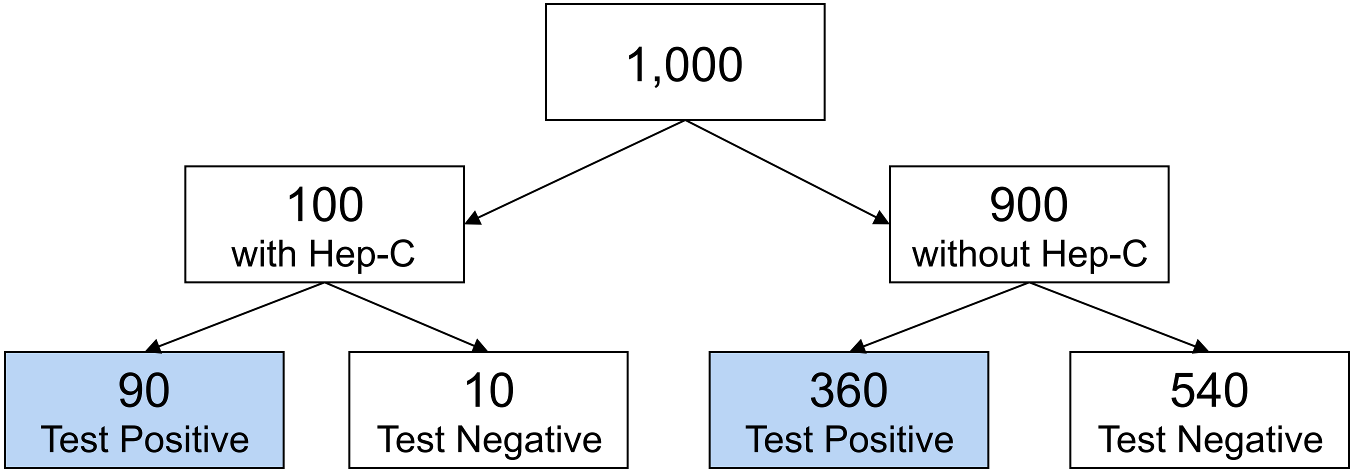


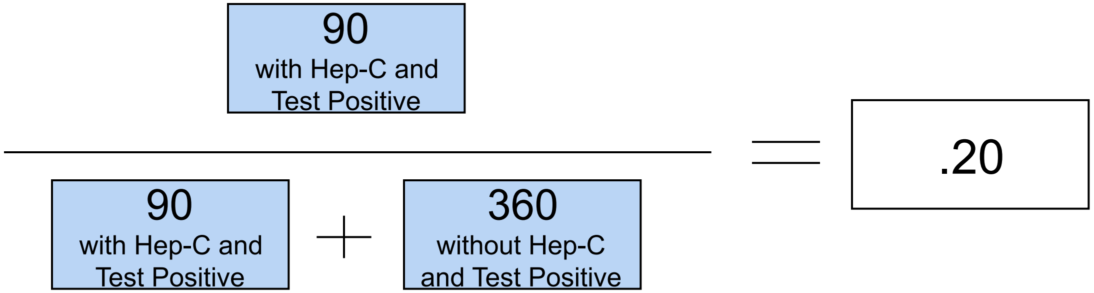

How does the correct value of 20% compare with the estimate that you provided at the start of the problem? If they differ, which one is greater and by how much?

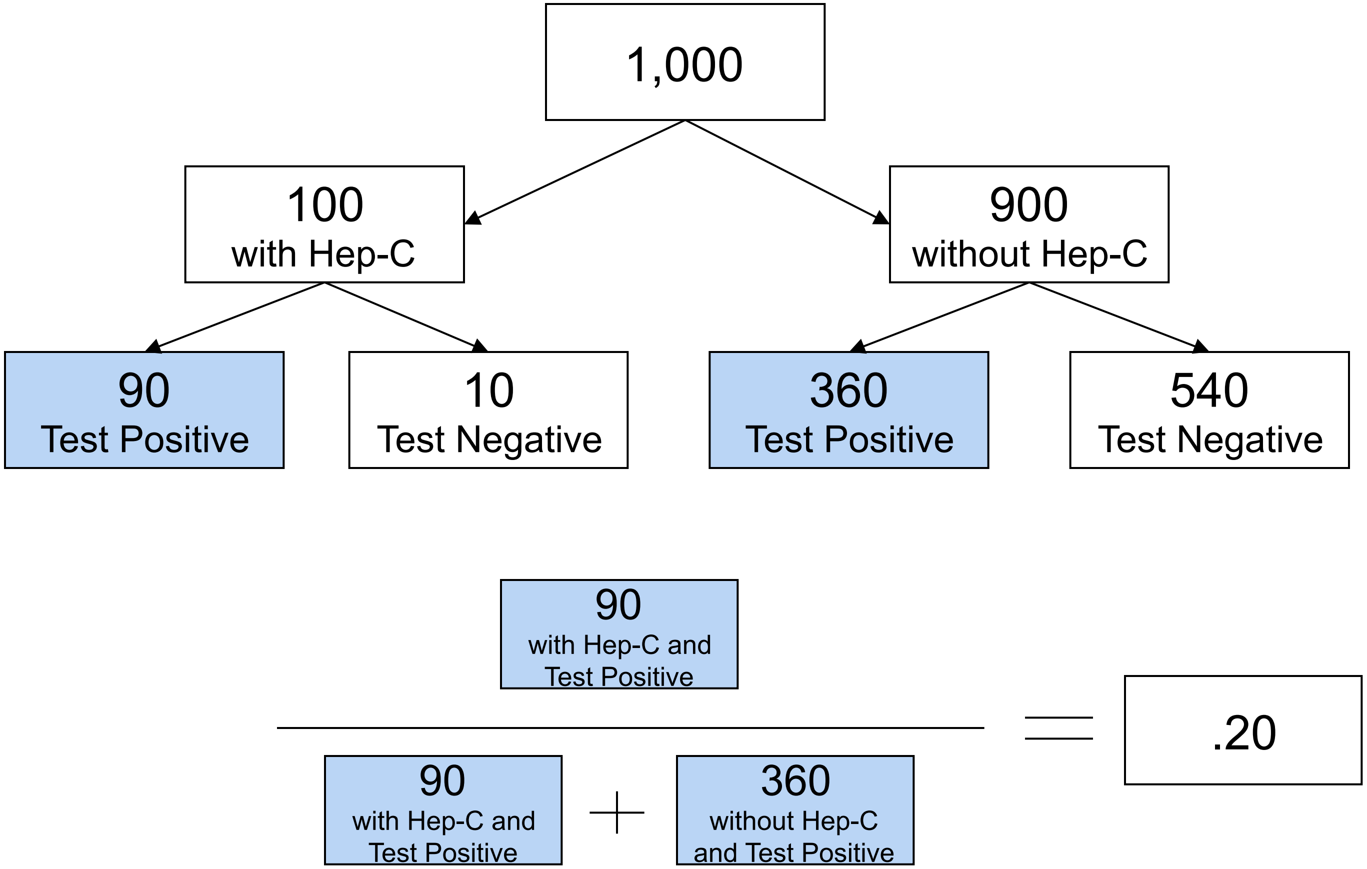

**Example 2: Different Medical Test Result**

Now that you' ve worked through the earlier medical problem, let' s consider how you would solve the problem if John had tested negative instead of positive on the medical test. Recall that you suspect that John might have Hep-C. As in the original problem, medical research indicates that Hep-C is found in 10% of the general population, and that 90% of people who have Hep-C test positive on the same test you gave to John. The research also indicates that 40% of people who do not have Hep-C also test positive on the same test due to the inaccuracy of the test.

Now if you wanted to assess the probability that John has Hep-C given that he tested negative, how would you go about doing that? Please begin by providing your initial estimate. Then we will work through a systematic way of solving this problem. Before advancing to the next screen, please write down your estimate in percentages in the booklet.

As in the first problem, we will now work through the solution step by step. Once again, we'll start with a random sample 1,000 people from the general population. Then, as we did before, let's divide this sample into the percentage of people who have Hep-C and the remaining percentage of people who do not have Hep-C. Since 10% of population have Hep-C, we expect that 100 of the 1,000 people will have Hep-C and the remaining 900 will not.

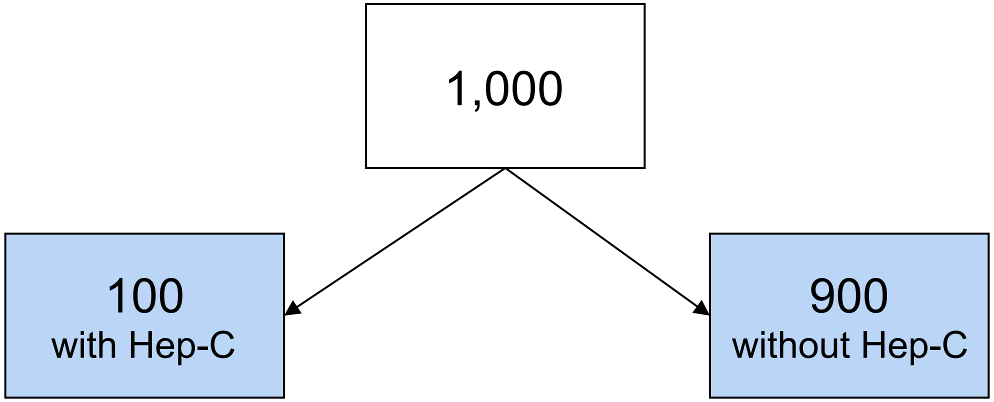

Next, we can break down each of the sub-samples into those who are likely to test positive and those who are likely to test negative. Since the medical statistics used in this problem are identical to those used in the initial problem, the breakdown is exactly the same and is shown graphically below:

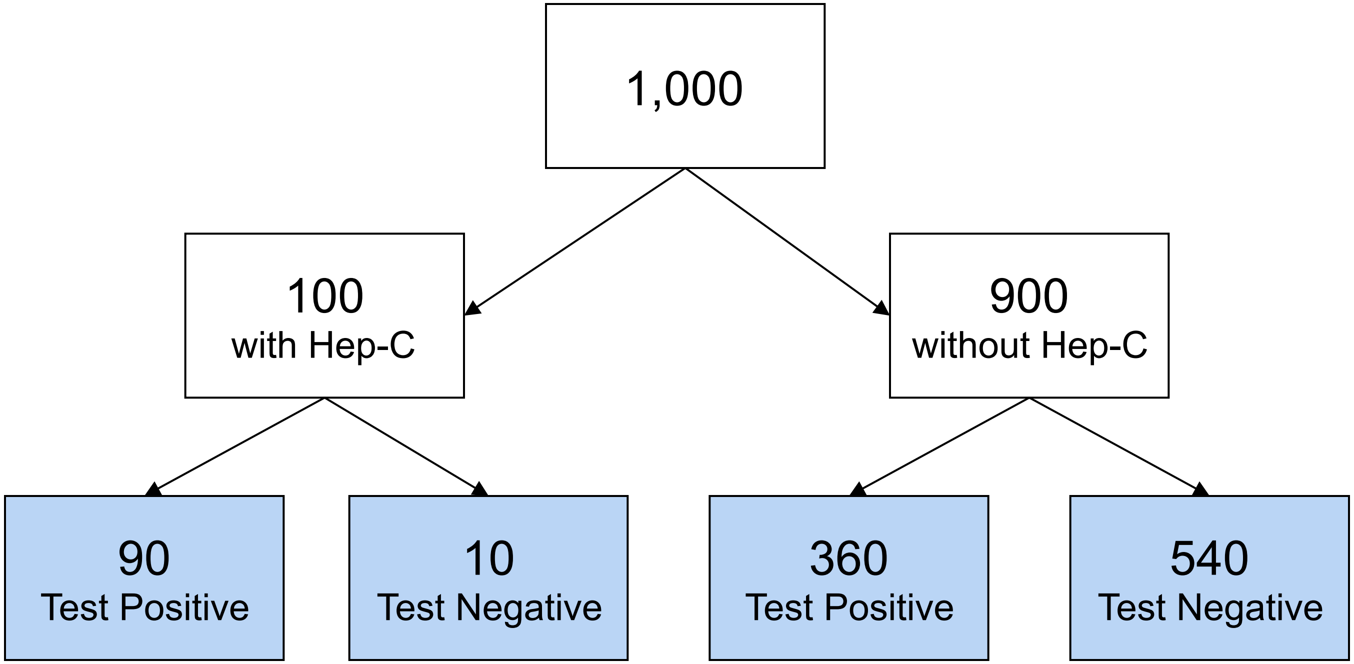

Now, the next question is what do we do with it in order to answer the question of interest—namely, *what is the probability that John has Hep-C given that he tested negative*?

First, we need to determine the number of people in our sample who are expected to test negative like John. To do so, we simply add the number of all people who test negative regardless of whether they have Hep-C (10) or not (540). These values sum to 550.

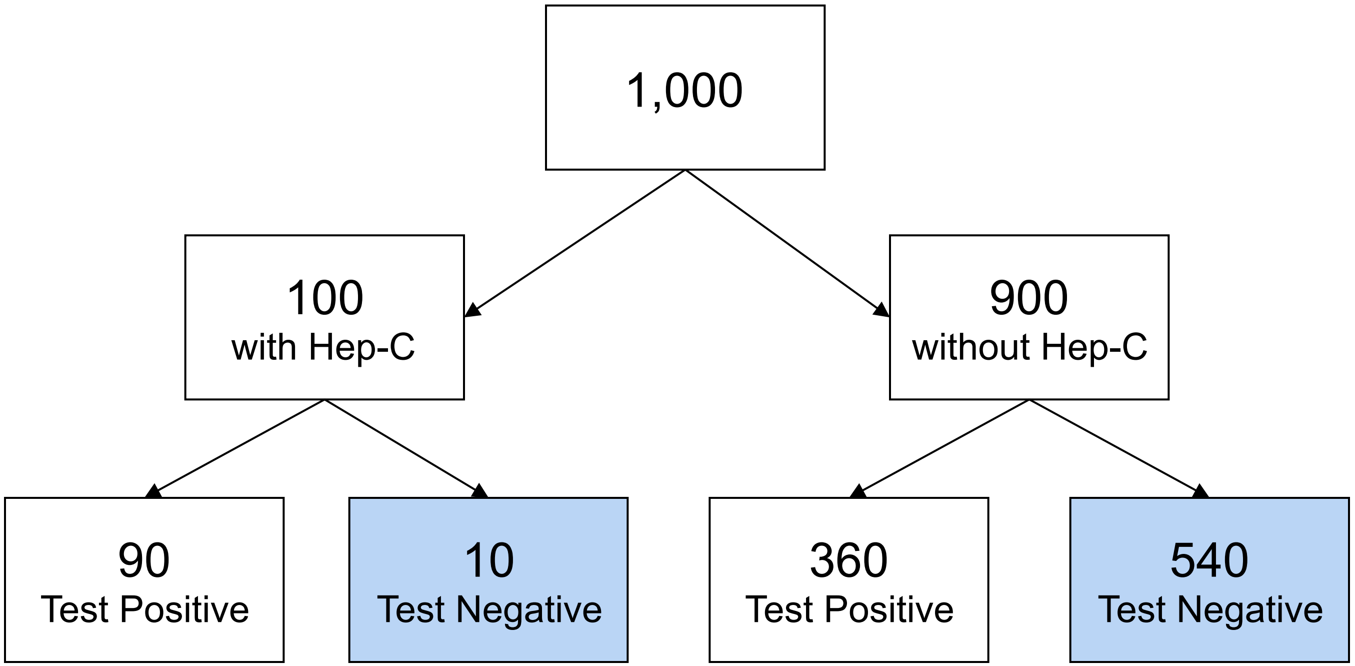

Now, to figure out the probability of John having Hep-C given that he tested negative, you will want to know how many out of those 550 people who test negative also have Hep-C. That ratio will be the answer to the question. Since we know that 10 of the 550 people who test negative have Hep-C, we simply divide 10 over 550 to get the answer. Since 10/550 = .02 (rounding to the nearest percentage point), there is about a 2% chance that John has Hep-C given his negative test result.

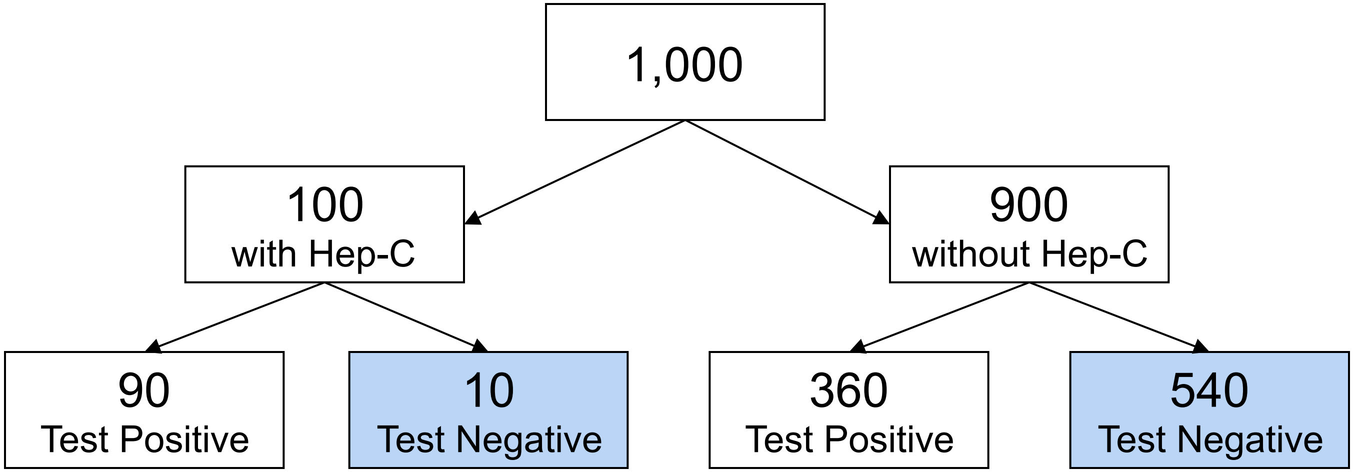


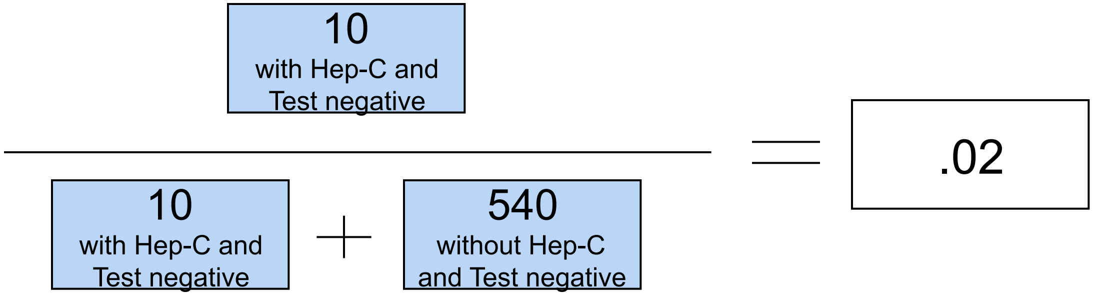

How does the correct value of 2% compare with the estimate that you provided at the start of the problem? If they differ, which one is greater and by how much?

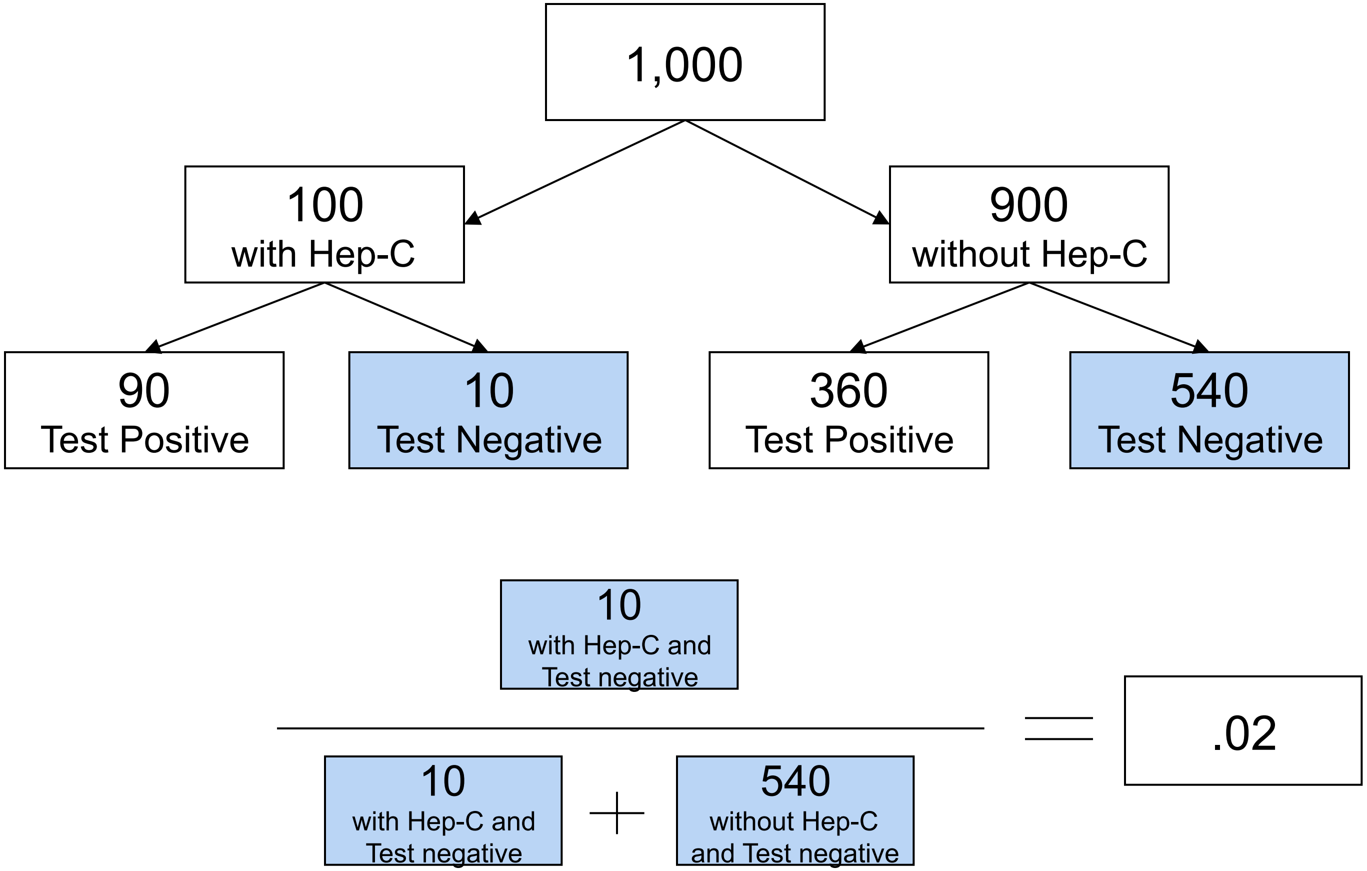

The step by step approach to estimating probability that you’ve just been shown can be used to answer several related questions. For instance, you started out by estimating the probability that John has Hep-C given that he had a positive test result (20% chance). Then, you estimated the probability that John has Hep-C given that he had a negative test result (2% chance). In the same manner, you could also answer the question “*What is the probability that John doesn’t have Hep-C given that he tested (let’s say) negative*?” Please try to work out the answer to this question on your own.

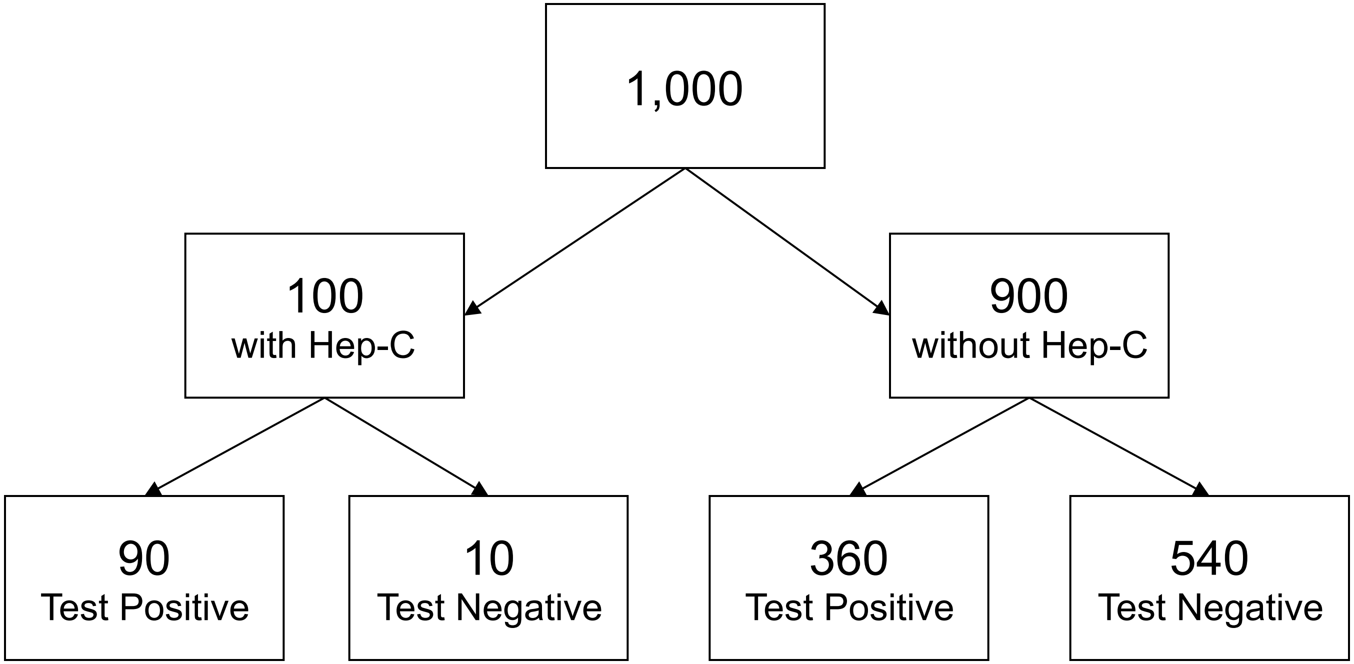

If you correctly followed the same approach taken in the first two problems, you would begin by finding out how many people tested negative. There are 550 people who test negative. Out of those people, you want to determine what percentage does not have Hep-C. There are 540 out 550 people who are expected not to have Hep-C and to test negative. Since 540/550 = .98, there is a 98% chance that John doesn't have Hep-C given that he tested negative. How does the correct value of 98% compare with the value that you provided? If they differ, which one is greater and by how much? How did you obtain your value?

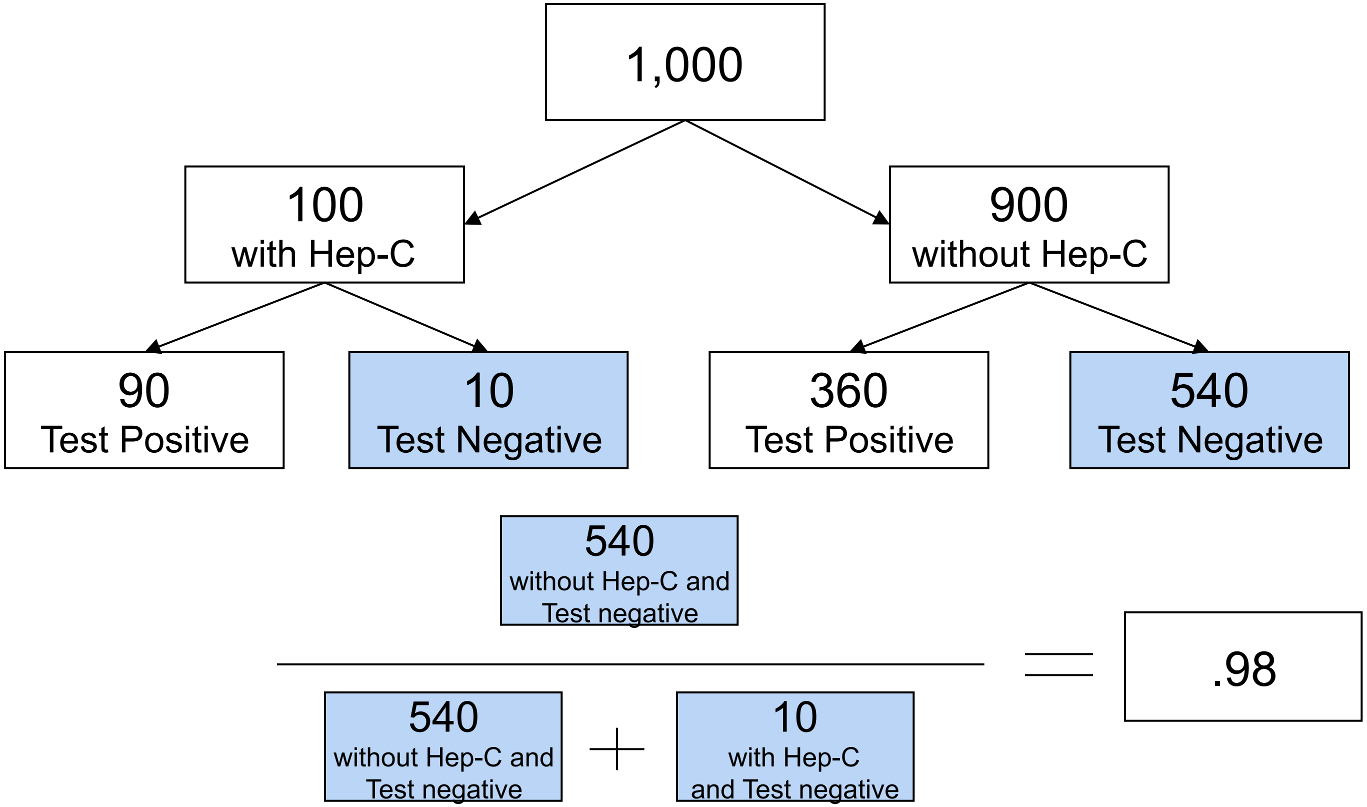

Notice that the answer to the last two questions – 2% and 98% -- add up to 100%. This is no coincidence. Since there are only two possibilities for people with a negative test result—namely, they either have Hep-C or they don't—if we know the probability of one of those possibilities, then we can easily obtain the other value simply by subtracting the first value from 100%. That is, if you know that the probability of having Hep-C given John tested negative is a 2% chance, then by subtracting 2% from 100% you can immediately know that the probability of John not having Hep-C given that he tested negative is 98%.

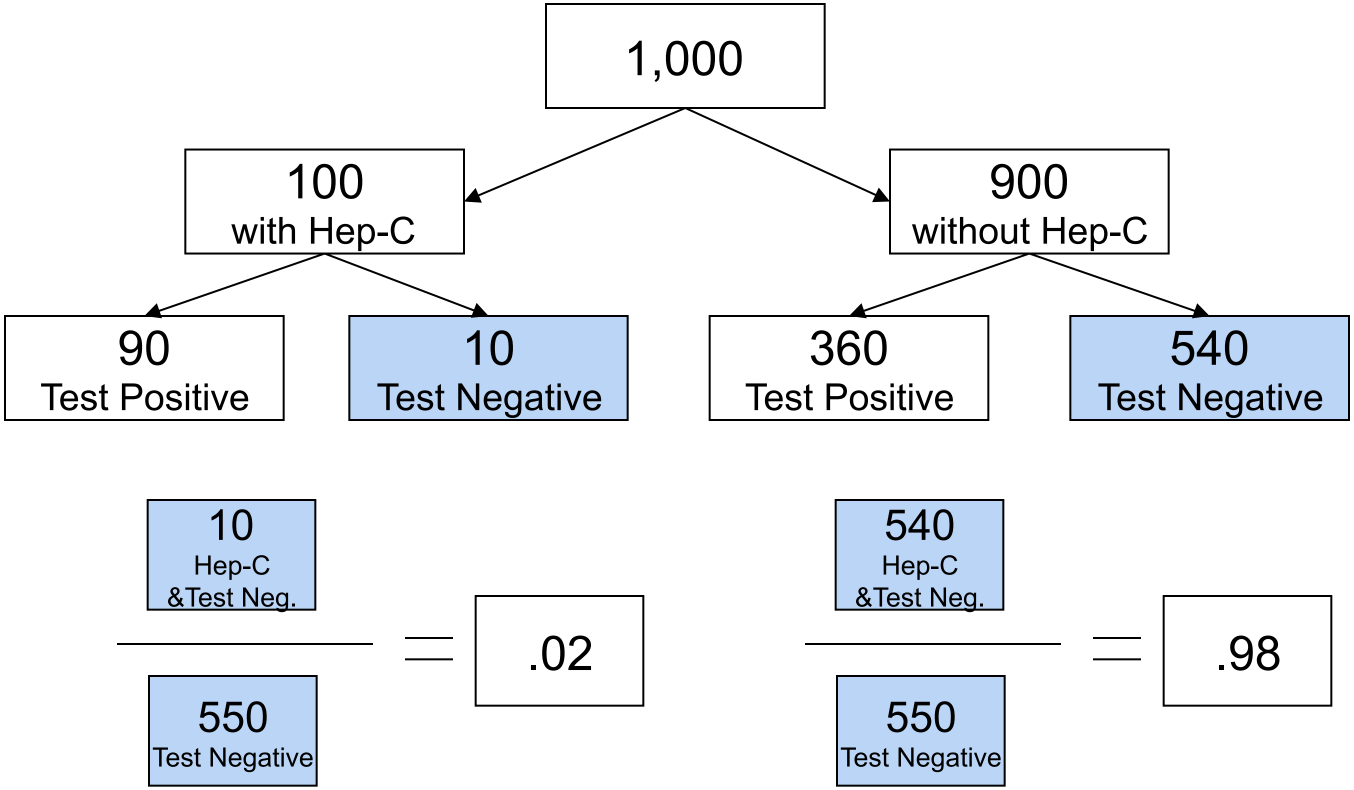

# Coherent assessments

Whenever you have two mutually exclusive and exhaustive hypotheses, such as (1) John has Hep-C and (2) John does not have Hep-C, the probabilities you assign to each should sum to 100%.

This logical principle is known as the *additivity property*. If your estimates violate the additivity property then you can be sure that they are not fully coherent. That is, they lack internal consistency, and the more they deviate from 100% when summed the more inconsistent they are.

For instance, imagine that you were assessing the probability that Group A was going to commit a terrorist attack somewhere in Canada in the next week and assessed the probability to be 1%.

Now imagine you were assessing the complementary probability that Group A was not going to commit a terrorist attack somewhere in Canada in the next week. If your assessments are coherent, you should assess the latter hypothesis to have a 99% chance of being true.

If you said, for instance, that the latter had an 80% chance of being correct, then your assessments would only add up to 81% and would be incoherent.

# Summary

- The aim of this tutorial was to assist you in approaching probability assessment tasks in a structured way that will lead to an accurate solution. Although you were presented with a lot of information in it, these essential points can be summarized as follows:

# Key Points

- First, when you’re provided with information that you need to consider in order to assess the probability of an event, assemble that information in a manner that makes the frequencies of all the possible subsets of events explicit. In this tutorial, that was accomplished using the box-chart diagrams. Organizing the information in this manner will reduce the chance of you forgetting to use a relevant piece of information. It will also help you at the “information integration” stage.

# Key Points

- Once the information is organized in an explicit manner, think about the probability that you are trying to assess as a ratio. If you can identify the relevant denominator and numerator, the rest is simple arithmetic (division, to be exact).
- Start with the denominator. In problems of the form "What is the probability of *X* given *Y*?", the denominator will be the sum of all *Y* cases, which can easily be found by consulting the box-chart you've constructed.

# Key Points

- Next, you have to decide what the appropriate numerator is. That will be the set of cases that include both *X* and *Y*. Accordingly, your numerator will always be a subset of your denominator (which also includes the set of Y cases that are not X cases).
- All that remains is to carry out the arithmetic (divide) and, if you followed these steps correctly, you will have the correct value of the probability you were trying estimate.

# The End

- That’ s it!
- Thanks for completing this tutorial.

- You’ ll now be asked to work through a new set of probability estimation tasks.
